# Structure–Property Relationships and Surface Engineering of Natural Biopolymers for Triboelectric Applications: The Role of Additive Manufacturing

**DOI:** 10.3390/polym18101260

**Published:** 2026-05-21

**Authors:** Patricia Isabela Brăileanu, Nicoleta Elisabeta Pascu, Tiberiu Gabriel Dobrescu

**Affiliations:** Department of Robotics and Manufacturing Systems, Faculty of Industrial Engineering and Robotics, National University of Science and Technology POLITEHNICA Bucharest, 060042 Bucharest, Romania; nicoleta.pascu@upb.ro (N.E.P.); tiberiu.dobrescu@upb.ro (T.G.D.)

**Keywords:** triboelectric nanogenerators, additive manufacturing, surface tribology, biomedical interfaces, cellulose, chitosan, silk fibroin

## Abstract

This comprehensive review aims to cover the surface tribology and triboelectric properties of additively manufactured (AM) natural biopolymers, including cellulose, chitosan (CS) and silk fibroin (SF), in biomedical interface engineering. While these sustainable materials exhibit innate biocompatibility and tribopositivity, their baseline triboelectric performance demands targeted surface engineering. We synthesize key physical mechanisms governing charge generation, emphasizing how controlled surface roughness, hierarchical porosity and nanoscale architectures maximize contact electrification. Furthermore, distinct dielectric and polarity modulation strategies are evaluated across the biopolymer families: cellulose relies heavily on chemical functionalization to overcome weak native polarity; chitosan utilizes ionic coordination and fillers to elevate its relatively low charge density; and silk fibroin achieves exceptional power outputs via highly porous three-dimensional nanocomposite aerogels. AM technologies afford unprecedented spatial control over these biointerfaces but introduce severe processing constraints. Techniques such as those based on extrusion impose strict shear-thinning rheology and rapid crosslinking for cellulose and chitosan, while SF frequently suffers from crystallization-induced nozzle clogging, necessitating photocurable derivatives.

## 1. Introduction

**Figure 1:**
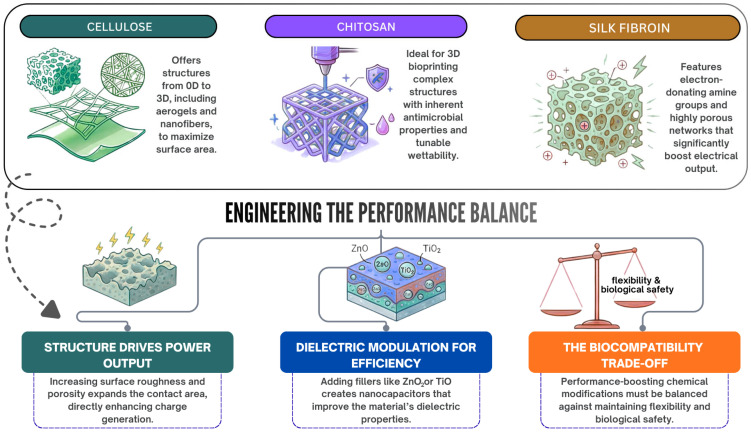
Overview of natural biopolymers and key surface-engineering principles for TENG performance.

Triboelectric nanogenerators (TENGs) are considered novel energy conversion systems that can be used for powering sophisticated electronics as well as electronic skins and self-powered wearable sensors. While standard TENGs generally rely extensively on inorganic materials and synthetic polymers, the fast-growing realm of biomedical bioelectronics calls for materials which not only should be highly efficient in terms of mechanical energy conversion but should also be biocompatible, environmentally friendly and easily degradable in the physiologic environment [1,2,3]. To address this, natural biopolymer materials such as cellulose, chitosan and silk fibroin have become a topic of much research due to their great potential as eco-friendly substitutes. They have intrinsic biocompatibility, excellent processibility and advantageous tribopositive properties, which render them well-suited for use directly in biomedical bioelectronic interfaces. A summary of the specific natural biopolymers under consideration, along with basic approaches in their surface modification, is shown in Figure 1.

To fully exploit the potential of these natural biopolymers, a rigorous understanding of their structure–property relationships is essential. The triboelectric behavior of a polymer interface is fundamentally governed by its molecular architecture, the density and polarity of its functional groups and its hierarchical morphology. For instance, the type and polarity of functional groups, together with their distribution within the polymer matrix, directly influence interfacial charge transfer behavior and the achievable surface charge density [1,2,3,4,5,6,7]. By rationally tuning these structural properties, such as modifying surface roughness, inducing controlled hierarchical porosity, or introducing dielectric fillers, researchers can directly modulate interfacial polarization, expand the effective contact area, and enhance charge generation efficiency [7,8,9]. Chemical modifications, ranging from functional group grafting to crosslinking, further alter the dielectric constant and trap density of the polymer matrix [8,9,10,11]. However, achieving a rational balance between enhancing these functional triboelectric characteristics and preserving the intrinsic biological and mechanical integrity of the biopolymer remains an important challenge in device design [8,9,10,11].

Translating these optimized macromolecular structures into functional biomedical devices relies heavily on advanced fabrication strategies. Historically, conventional processing methods, such as solution casting, electrospinning and freeze-drying, have been widely utilized to manufacture continuous films, fibrous membranes and highly porous aerogels [2,3,12,13,14,15,16]. While these traditional techniques are effective for generating bulk porosity or two-dimensional textured surfaces, they generally offer limited control over complex, three-dimensional (3D) macrostructures and precise micro-architectures. In contrast, additive manufacturing (AM) represents an advanced fabrication approach that enables enhanced geometric and architectural control compared to conventional techniques. Characterized by digitally controlled, layer-by-layer deposition, AM provides unprecedented, deterministic control over geometric design [3,7,17,18,19,20,21]. This approach enables precise spatial patterning of hierarchical micro-architectures, tunable internal porosity and the fabrication of application-specific geometries [9,22,23,24,25]. Consequently, AM can enhance triboelectric interface design compared to conventional methods by enabling tailored surface topographies that promote efficient contact electrification. However, the inclusion of biological polymers in AM systems brings its own set of rigid requirements for the materials. Rheological characteristics like shear-thinning and yield stress need to be controlled while preparing effective bioinks. Rapid post-printing stabilization is also necessary to maintain shape fidelity [1,3,7,12,13]. These processing constraints strongly influence the final structural, mechanical and electrical properties of the additively manufactured structures.

While previous reviews have discussed TENGs and bio-based materials, limited attention has been given to the relationship between polymer structure, surface engineering, AM and triboelectric performance in biomedical applications. Therefore, this review provides an integrated perspective focused on structure–property relationships and the role of AM in tailoring triboelectric biointerfaces.

Within this conceptual framework, the present review provides a comprehensive and comparative analysis of three representative natural biopolymers, cellulose, chitosan and silk fibroin, with particular emphasis on the role of additive manufacturing in enabling advanced structural and surface control for triboelectric applications. This review systematically explores the interplay between polymer structure, surface engineering, dielectric modulation and processing routes. By delineating fundamental structure–property relationships and clearly distinguishing the capabilities of AM from conventional fabrication techniques, this work aims to elucidate the core design principles governing triboelectric performance. Ultimately, this review highlights the inherent trade-offs among electrical performance, biocompatibility and mechanical stability, identifies current methodological limitations and outlines future directions for the rational design of sustainable, high-performance triboelectric biointerfaces.

## 2. Materials and Methods

To comprehensively evaluate the surface tribology and triboelectric behavior of AM natural biopolymers for biomedical interface engineering, an extensive literature search was conducted. The primary electronic databases utilized for this review included Web of Science, Scopus, PubMed and Google Scholar. The search strategy employed a combination of targeted keywords to effectively capture the relevant literature at the intersection of biopolymers, advanced manufacturing and energy harvesting. Specific search terms incorporated “triboelectric nanogenerators” or “TENGs,” alongside “cellulose,” “chitosan,” “silk fibroin,” “surface tribology,” “additive manufacturing,” and “biocompatibility.” Boolean operators (“AND”, “OR”) were applied to refine query combinations, and reference lists from key articles were manually screened to identify additional relevant sources.

Approximately 900 publications were initially identified through database searching. After duplicate removal, approximately 700 records remained for preliminary title and abstract screening. Around 400 studies were subsequently evaluated for relevance to triboelectric nanogenerators, natural biopolymers, surface engineering and additive manufacturing. Following full-text assessment and application of the predefined inclusion and exclusion criteria, approximately 210 highly relevant peer-reviewed studies were retained for qualitative synthesis and comparative analysis within the scope of this review.

Only articles published between 2000 and 2026 were considered eligible, reflecting the period in which triboelectric nanogenerators and bio-based interface engineering technologies have gained significant scientific attention. The scope of this review was strictly defined to focus on three specific natural biopolymers: cellulose, chitosan and silk fibroin. To be eligible for inclusion, articles were required to be peer-reviewed studies that prioritized investigations into the triboelectric or surface-related behavior of these specific biomaterials. Additionally, the selected studies were required to involve either additive manufacturing approaches or conventional fabrication techniques commonly used for comparison with AM-derived triboelectric structures.

**Figure 2:**
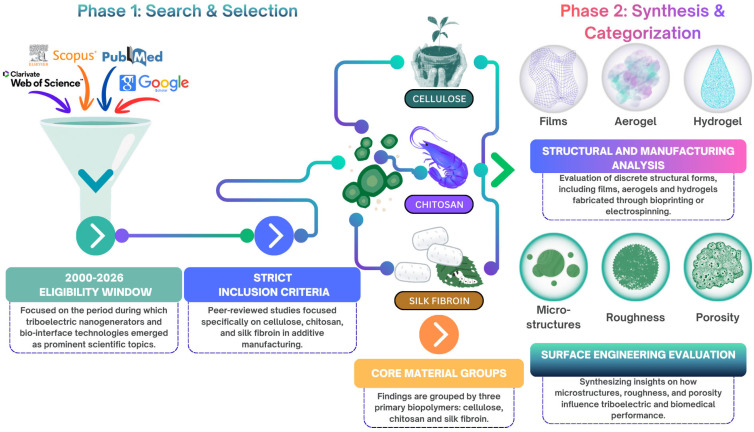
Overview of the two-phase review methodology, including the search and selection strategy and the subsequent synthesis and categorization of studies.

The exclusion criteria generally comprised non-peer-reviewed relevant reports, conference abstracts, patents, studies lacking triboelectric or surface-engineering data and works that exclusively addressed synthetic polymers without a natural biopolymer component. Figure 2 provides a schematic overview of the two-phase review methodology applied in this study.

The selected publications were systematically categorized according to material type, fabrication strategy, structural morphology and surface-engineering approach in order to identify recurring trends and structure–property relationships relevant to triboelectric performance. Studies were primarily grouped according to the specific material type (cellulose, chitosan and silk fibroin). Across these overarching material groups, the literature was further sub-categorized based on the processing methods utilized. This review aimed to integrate the fabrication of discrete structural forms, such as films, fibers, aerogels and hydrogels, as well as distinct manufacturing techniques, including electrospinning, freeze-drying and extrusion-based bioprinting. The synthesized data obtained were evaluated based on essential surface-engineering strategies (e.g., the modulation of micro/nanostructure, surface roughness, porosity, dielectric properties and functionalization) and their influence on both triboelectric outcome metrics and overall biomedical interface performance. A quantitative meta-analysis was not performed due to substantial heterogeneity in experimental configurations, material formulations, device architectures and reporting methodologies across the selected studies.

## 3. Cellulose-Based Triboelectric Biopolymers

### 3.1. Processing–Structure Relationships in Cellulose-Based TENGs

The structure of cellulose involves a polysaccharide chain polymer composed of glucose units [26,27,28]. Also, the hydroxyl groups present on these units facilitate the formation of hydrogen bonds between chains, leading to the development of sturdy nanofibril and microfibril structures with distinguished mechanical strength [26,29,30,31,32]. The length or molecular weight of the cellulose chain is predominantly dependent on its source or the processing method employed, as highlighted by Zhang et al. [26]. Cellulose can be found in nature in several forms, including microcrystalline cellulose (MCC), cellulose nanofibrils (CNFs) and cellulose nanocrystals (CNCs), which are often extracted from lignocellulosic biomass or produced by bacteria as bacterial cellulose (BC) [27,33,34,35,36,37,38,39]. Troncoso et al. stress that lignocellulosic biomass itself is mainly composed of cellulose (35–50%), lignin (5–30%) and hemicellulose (20–35%) [34]. Generally, pure cellulose is obtained by removing other components like hemicellulose and lignin [34].

Cellulosic materials can be processed using various methods, some of which fall under or are related to AM and film fabrication techniques. Zhou et al. highlights that nanofiber films can be manufactured as triboelectric layers using methods like electrospinning and a fast solution blowing method [12]. Cellulose II aerogels can be developed through a combination of dissolution–regeneration and freeze-drying [1,12]. Paper represents one of the most accessible and easily processable cellulose-derived materials employed in triboelectric nanogenerators [1]. Functionalized native wood surfaces have been created, as outlined by Du et al. [1]. Cellulose nanofibril (CNF) films, for example, can be synthesized [1]. Hydroxyethylcellulose (HEC) and porous nitrocellulose are also important candidates for TENGs [1].

Cellulose II aerogel, reported by Zhou et al., features a special 3D nanostructure, distinct from 2D film structures [12]. This aerogel also has abundant mesopores, a larger surface area and an internal uniform continuous network structure [1,12]. CNF films can be modified, for example, with aminosilane, enhancing hydrophobicity. Du et al. describe how the surface potential of CNFs can be tailored by adjusting the number and density of functional groups [1].

Bacterial cellulose (BC) films can be fabricated by gradually drying BC hydrogel on a substrate [39]. BC/ZnO nanocomposites can have ZnO nanoparticles impregnated into the BC nanostructure, particularly at the film surface, to increase surface roughness [39]. As noted by Jakmuangpak et al., controlling micro–nano structure is a method to enhance the output performance of bacterial cellulose nanofiber-based TENGs [39]. Cellulose acetate can be modified with ZnO or TiO_2_, influencing surface roughness based on the homogeneity/aggregation of doping agents, as reported in Candido et al.’s study [40].

Fabrication strategies employed for cellulose-based materials critically determine the resulting microstructural architecture, interfacial topography and mechanical deformability of the matrix, all of which play a central role in regulating the triboelectric response of the system. For instance, freeze-drying generates highly porous compressible architectures that improve contact-separation dynamics, while electrospinning produces interconnected nanofibrillar networks with high specific surface area and enhanced frictional contact efficiency [1,12]. Similarly, nanoparticle incorporation and surface functionalization modify roughness, dielectric heterogeneity and surface potential, thereby affecting charge transfer behavior and output stability during cyclic mechanical loading [39,40]. Table 1 below presents a comparative account of the important cellulose-based triboelectric systems, which brings into light the interplay between the method of fabrication, structure, physicochemical properties and functionalities.

The literature reveals a profound interdependence between the processing pathways, the resulting structural morphologies, and the ultimate triboelectric performance of cellulose-based systems [42,47]. Processing strategies sequentially traverse multiple length scales, ranging from macroscopic physical modifications to molecular-level chemical tailoring, to overcome the intrinsically weak tribo-polarity and low dielectric constant of pure cellulose [47,48]. Extrusion-based AM (e.g., direct ink writing) and electrospinning techniques usually dictate the geometric arrangement of fibrillar networks, yielding highly deformable, hierarchically porous matrices that mechanically amplify the effective interfacial contact area during cyclic compression [42,43,44]. Concurrently, thermodynamic regeneration, template-assisted molding and mechanical creping modulate topographical compliance and structural elasticity, enabling intimate conformal contact against counter-layers [41,45]. Beyond physical architecture, the integration of high-*k* and conductive nanofillers establishes dense microcapacitor networks, which considerably induce interfacial polarization and optimize the equivalent capacitance while carefully managing percolation threshold limitations [1,42,46]. Advanced surface engineering, such as fluorination, amination or sulfonation, directly intervenes in the local electronic band structure, substantially shifting the surface potential phase and engineering electrostatic deep traps [1,4]. This molecular-scale intervention effectively suppresses the kinetic dissipation of triboelectric charges [4]. Therefore, the synergistic modulation of macroscopic topographical deformability, interfacial dielectric polarization and molecular charge-trapping kinetics positions customized cellulose architectures as attractive platforms for high-performance, sustainable mechanosensing and energy harvesting systems.

Cellulosic materials are described in the scientific literature as biocompatible [1,39,49,50,51,52,53]. CMC is noted as non-toxic to the body and environmentally friendly by Zhou et al. [12]. According to Wang et al., some methods to increase triboelectric charge density, such as certain chemical modifications, surface patterning, dipole orientation and structural optimization, can dramatically reduce the materials’ mechanical strength, flexibility, biocompatibility and transparency [4]. Accordingly, balancing dielectric enhancement with properties like mechanical strength and biocompatibility is a challenge [4].

**Figure 3:**
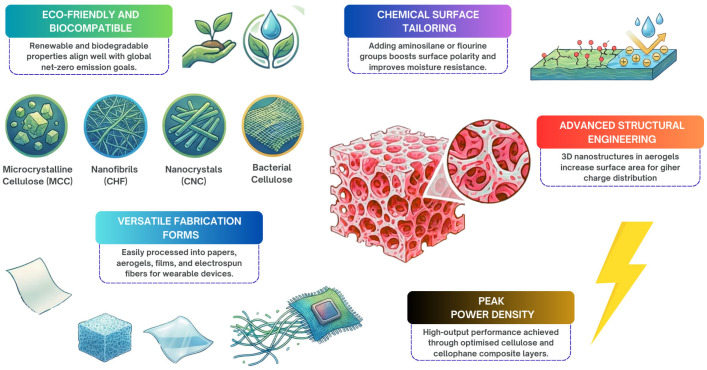
Cellulose-based system’s main characteristics for TENGs.

These materials are widely investigated for their application as triboelectric layers in TENGs, and cellulosic materials can function as either electron-giving or electron-losing components within a TENG system [26,40,54]. Various forms of cellulose are employed, including paper, wood, aerogels, films and fibers [1,26]. Paper, being simple to manufacture and cost-effective, is a common cellulosic triboelectric material used in basic TENG structures as shown in Du et al.’s study [1]. Cellulose aerogels are valued for their low density, high porosity and large specific surface area, contributing to enhanced triboelectric performance [1]. Cellulose nanofiber films can also serve as effective triboelectric layers [12]. Some studies report considerable output performance from cellulose and cellophane, achieving power densities up to 300 W m^−2^ (Du et al.) [1,40,55]. An overview of the major properties concerning cellulose-based TENGs can be seen in Figure 3.

Overall, cellulose and its derivatives offer significant advantages for environmentally friendly and biocompatible TENGs, including renewability, biodegradability, low cost and suitability for wearable and biomedical applications [12,26,33,34,35,40,52,54]. They can be formed into various structures like films, fibers and aerogels [1,12]. The primary limitation is the weak intrinsic triboelectric polarity of pure cellulose [54]. However, this can be effectively overcome through surface modifications, chemical functionalization, incorporation of fillers and control over microstructure, porosity and fibril arrangement [1,33,40,48,54].

### 3.2. Additive Manufacturing Approaches for Cellulose-Based TENGs

Cellulose has a material selection space from 0D to 3D with different dimensions and morphologies [1,28]. To fully exploit this multidimensionality in the design of next-generation energy harvesting technologies, AM has emerged as an impactful approach. Unlike conventional film-casting or molding techniques that often result in homogenous, planar substrates with limited interfacial design freedom, AM enables the controlled engineering of hierarchical structures, adjusted porosity and precise spatial distribution of functional additives [56]. By linking the gap between molecular-level cellulose chemistry and macroscopic device architecture, AM facilitates the fabrication of cellulose-based TENGs with significantly adjustable mechanical resilience and triboelectric performance [43,57].

The feasible implementation of AM in cellulose-based TENGs relies mainly on the development of printable cellulosic formulations with rigorously controlled rheological properties. Extrusion-based technologies, particularly direct ink writing (DIW), require viscoelastic inks that exhibit pronounced non-Newtonian shear-thinning behavior [44,56]. During the extrusion process, the applied shear force must sufficiently lower the apparent viscosity (where the loss modulus dominates) to ensure smooth flow through a micro-nozzle, followed by an instantaneous structural recovery (where the storage modulus dominates) upon deposition to prevent pattern collapse [44,56]. Achieving this rheological balance with pure cellulose suspensions often presents a comprehensible challenge due to the strong intermolecular hydrogen bonding and high degree of polymerization associated with nanocellulose, which can lead to nozzle clogging or poor shape fidelity [56,57]. To help overcome these issues, researchers have increasingly turned to cellulose derivatives and composite inks. Carboxymethyl cellulose (CMC) has been used in 3D printing, where hexagonal boron nitride (hBN) was added as a rheology modifier to enhance its printability, as reported by Jayakumar et al. [49,56]. In this context, hBN acts not only as a viscosity regulator to facilitate smooth layer-by-layer deposition but also substantially improves the thermal stability, mechanical strength and flexoelectric polarization of the printed CMC matrix, rendering it quite efficient for strain-induced charge generation [49]. Likewise, very concentrated CNC inks are favored for their rigidity and geometric aspect ratio, offering reliable self-supporting features while concurrently serving as high-strength piezoelectric/triboelectric active phases in 3D-printed architectures [57]. Table 2 comparatively summarizes the principal AM strategies employed for cellulose-based TENG architectures, emphasizing the relationship between printable formulation design, rheological behavior, structural programmability and triboelectric functionality.

AM fundamentally diverges from conventional film-casting or template-molding techniques by allowing consistent, bottom-up spatial control over the internal architecture and interfacial morphology of cellulose-based TENGs. While conventional processing typically yields planar, homogenous substrates that notably restrict structural compliance, AM routes, particularly DIW and electrohydrodynamic processing, help create hierarchical pore structures, programmable structural deformability and multiscale roughness [43,61].

A critical trade-off in extrusion-based AM lies in rheological optimization because cellulosic inks must exhibit pronounced non-Newtonian shear-thinning for smooth micro-nozzle extrusion yet demand near-instantaneous viscoelastic recovery to prevent the gravitational collapse of printed 3D hierarchical grids [43,56]. In addition, incorporating conductive or high-*k* fillers (e.g., CNTs, hBN) for dielectric modulation introduces rigid processing constraints. While elevated filler loadings mechanistically enhance interfacial polarization and triboelectric charge transfer, they proportionally increase the risk of nozzle clogging and seriously compromise continuous printability [44,49]. Meanwhile, electrohydrodynamic printing is effective at generating ultra-porous nanofibrous webs with extreme specific surface areas that amplify contact electrification, yet it intrinsically lacks the macroscopic 3D geometric control afforded by DIW [60,61]. Despite the particular structural advantages introduced by AM, such as amplified mechanical compressibility and maximized effective contact areas, scalability and reproducibility remain notably constrained by complex post-print drying dynamics (e.g., freeze-drying), which frequently induce anisotropic volumetric contraction and dimensional distortion [43]. Overcoming these viscoelastic and post-processing limitations is important for transitioning AM-fabricated cellulosic TENGs from laboratory-scale prototypes to commercially viable, self-powered smart systems.

Beyond facilitating complex geometries, the primary advantage of AM in TENG fabrication is the ability to carefully engineer the interfacial contact area and structural deformability. AM techniques, such as DIW printing of ethyl cellulose and carbon nanotube inks on cellulose paper for electrodes, are addressed in the study by Shi et al. [44], demonstrating the feasibility of ink-based printing for specific components. By directly writing conductive CNT or metallic networks into ethyl cellulose or CNF matrices, researchers can construct 3D continuous conductive pathways that modulate the dielectric properties of the device [35,44,54]. This dielectric modulation is necessary because an optimized dielectric constant directly increases the existing capacitance of the triboelectric layer, thereby boosting the surface charge density and accelerating electron transfer during contact-separation cycles [1].

It should also be noted that DIW allows precise architectural design of hierarchical micro/nano-patterned cellulose aerogels. For instance, by printing alternating layers of CNF hydrogels with specifically programmed tilt angles and mesh sizes, subsequent freeze-drying produces a 3D-patterned aerogel with particular porosity and structural compressibility [43]. This macroscopic structural elasticity ensures that, under equivalent mechanical stimulus, the grid-like cellulose framework undergoes significant volumetric deformation, markedly amplifying the effective triboelectric contact area with the counter-electrode [43]. Thus, such all-printed 3D CNF TENGs have demonstrated electrical outputs that substantially surpass their conventionally molded, non-patterned counterparts, providing sufficient power to drive arrays of light-emitting diodes and function as highly sensitive self-powered biomechanical sensors [43].

An additional phenomenon specific to extrusion-based AM is the shear-induced alignment of anisotropic cellulosic particles. As the highly viscous ink is forced through the narrow printing nozzle, shear forces induce the longitudinal alignment of CNCs and CNFs along the printing direction [58,59]. Once regeneration or solvent evaporation occurs, this very ordered, closely packed nanofibrillar orientation yields printed structures with promising anisotropic mechanical properties, significantly improving the tensile strength, toughness and cyclic durability of the TENG device [58,59]. This structural stability is important for wearable TENGs, which must withstand thousands of continuous bending and compression cycles without suffering microstructural fatigue or loss of triboelectric performance.

In parallel to extrusion methods, electrohydrodynamic processes offer another critical avenue for structural engineering. Also, electrospinning is frequently used to produce cellulose nanofibers for TENGs [58,59,61]. Although it is not typically classified as 3D printing, electrospinning is a process that builds structures layer by layer. This technique performs well in the fabrication of ultra-porous, nonwoven nanofibrous webs with particular surface-area-to-volume ratios, a relevant requirement for maximizing contact electrification [60,61,62,63]. The integration of cellulose derivatives via multi-fluid electrospinning allows for the synergistic blending of tribopositive and tribonegative materials. For instance, the co-electrospinning of CEC or cellulose acetate CA with piezoelectric polymers like polyvinylidene fluoride (PVDF) significantly reduces the fiber diameter while enhancing surface roughness [60,61,63]. The high specific surface area and structural porosity not only trap more static charges but also increase the mechanical flexibility and breathability of the resulting TENG, making electrospun cellulosic membranes appropriate candidates for wearable respiratory sensors, air filtration energy harvesters and smart electronic textiles [60,61].

Despite these remarkable advancements, the widespread implementation of 3D printing for cellulosic TENGs faces notable scalability and processing limitations. While specific AM processing techniques for complete cellulose TENG fabrication remain less explored than conventional methods, DIW printing indicates growing interest in tailored cellulose-based triboelectric devices [44,54,59,62]. One of the main challenges is dealing with severe and often anisotropic volumetric shrinkage that occurs during the post-printing drying or solvent-exchange phases, particularly for hydrogel-based cellulose inks [56]. This contraction can compromise the shape fidelity and dimensional accuracy of the rigorously designed micro-patterns, inadvertently altering the predicted triboelectric contact mechanics. At the same time, while AM performs well in creating macro- and microscale architectures, achieving the requisite secondary nanoscale roughness, which is useful for maximizing triboelectric charge density, often necessitates hybrid approaches. Combining 3D printing with secondary treatments, such as in situ polymerization of conductive polymers (e.g., polypyrrole), chemical vapor deposition of silanes for hydrophobicity, or post-print freeze-drying, adds complexity to the manufacturing pipeline but remains necessary to achieve ultrahigh-output performances [44,61].

Other developments in cellulose-based TENGs could focus on harnessing the complete capabilities of multi-material and multi-method AM. The development of advanced, solvent-free or UV-curable cellulosic resins could reduce current contraction issues and accelerate printing speeds. Also, the seamless co-printing of triboelectric active layers, highly conductive cellulosic electrode networks and porous dielectric spacers in a single, continuous manufacturing step represents the primary objective for scalable self-powered systems. The ability to customize surface properties and structures through AM-compatible methods positions cellulose as a promising material for high-performance, sustainable and biocompatible TENGs [1,35,56,58]. Taken together, as AM technologies and rheological formulations mature, the predefined control over the 3D architecture of cellulose will apparently drive the transition of green TENGs from laboratory prototypes to commercially viable, self-powered smart electronics and ubiquitous environmental energy harvesters.

### 3.3. Surface Engineering and Dielectric Modulation of Cellulose-Based TENGs

To overcome the relatively weak surface polarity of pure cellulose compared to synthetic polymers, engineering surface texture and porosity in cellulose-based materials is a determining factor for enhancing triboelectric performance, particularly in biocompatible applications like wearable and implantable electronics [39,54,63]. Various techniques are employed to tailor these surface features [1]. Methods include dissolution–regeneration combined with freeze-drying to produce cellulose II aerogels, which possess a special 3D nanostructure where induced charges distribute throughout the structural network, not just the immediate contact surface, as reported by Zhou et al. [12]. Solution blowing can yield fluffy and porous nanofiber membranes [12], while electrospinning is also used to prepare cellulose nanofibers and derivatives like cellulose acetate nanofibers [35,40,44]. AM techniques such as 3D printing and DIW facilitate the creation of customized structures, porous scaffolds and micro/nanostructures in cellulose and its composites [49,54,64].

Enhancing the triboelectric performance of cellulose materials is an active area of research, with a significant focus on modifying their surface texture, porosity and chemical properties [1,48,54]. Surface modification is a trending strategy to boost the surface polarity and the number of functional groups, thereby increasing the output performance of TENGs. For instance, aminosilane modification of CNF films has been shown to enhance positive polarity and generate numerous surface charges [1]. This treatment also confers hydrophobicity, improving moisture resistance [1]. Conversely, incorporating fluorine-containing groups can improve the electron-withdrawing ability, leading to hydrophobic negative triboelectric materials [1]. Therefore, the density and type of functional groups can be precisely tailored to control the surface charge density of cellulose nanofibrils [1].

Beyond chemical modification, altering surface morphology and structure is important for improving charge production through electrostatic induction and contact electrification [54]. In Cho et al.’s study, techniques like micro/nanopatterning, imprinting lithography, spin coating, electrospinning and etching have been used to alter surface morphologies, effectively improving the output performance of cellulose-based TENGs [54]. For example, porous and fluffy films prepared by solution blowing can yield higher voltages compared to electrospun films [12]. Cellulose aerogels, with their special 3D nanostructure and abundant mesopores, offer a larger surface area and allow induced charges to distribute across the structural network, not just the contact surface [1]. Hierarchical and porous structures, particularly in aerogels or thin films incorporating 2D fillers, are advantageous for applications like gas sensing TENGs as they create more gas capture sites [1]. Composite materials can be designed with hierarchical structures rich in interfacial regions to induce strong interfacial polarization or layered porous structures to provide loading sites for conductive materials like carbon nanotubes or PANIs [1,65,66]. Incorporating conductive fillers such as carbon black (CB), carbon nanotubes (CNTs), or graphene nanoplatelets (GNPs) into cellulose matrices like MCC or CNF can significantly influence electrical properties and enhance output voltage in triboelectric mode [33,55]. Factors like the specific surface area of the filler, its nanoscale dispersion, filler content and matrix porosity can have a stronger influence on the triboelectric voltage than the filler’s conductivity alone, as highlighted in González et al.’s study [33]. Adding components like polyamide (PA6), polyvinylidene fluoride (PVDF) and barium titanate (BaTiO_3_) to regenerated cellulose membranes has been shown by Sun et al. to substantially increase output voltage and current, alongside altering the surface morphology [67]. A cellulose-based TENG incorporating ZIF-8 and MO-PPy analyzed by Li et al. demonstrated enhanced performance, achieving a transfer charge of 47.4 nC, an open-circuit voltage of 129 V and a short-circuit current of 6.8 µA, which was about four times higher than a similar material without MO-PPy [68].

Surface modification by incorporating nanoparticles (e.g., ZnO, carbon black, Fe_3_O_4_), carbon nanotubes or silanes is also effective [1,33,39,40,46,55,68]. Impregnating ZnO nanoparticles into bacterial cellulose increases surface roughness and polarizability [39]. These modifications and porous structures generally increase the available surface area for charge accumulation, enhance contact efficiency, tune surface polarity and can introduce dielectric effects from fillers [1,40,46,63]. For instance, ZnO-doped cellulose acetate membranes demonstrated significantly improved output, achieving up to 282.8 V, 3.42 µA and a power density of 60 µW/cm^2^, outlined by Candido et al. [40]. Jakmuangpak et al. evidenced how a bacterial cellulose/ZnO TENG reached 57.6 V, 5.78 µA, and 42 mW/m^2^ [39]. Increased surface roughness (e.g., from 127 nm to 195 nm after modification) and altered surface potential can correlate directly with enhanced triboelectric output, as noted by Zhu et al. [69]. Porous conductive wood-based materials have also shown ability to generate additional triboelectric charges [1]. While surface engineering improves performance, these techniques can add complexity and cost [54]. Nevertheless, the natural biodegradability and biocompatibility of nanocellulose provide unique advantages for sustainable biomedical TENGs [63].

Increased surface roughness is believed to enhance TENG performance by increasing the contact area, thereby improving friction and charge generation [8,40]. Dielectric modulation of cellulose is mandatory for increasing the surface charge density [1,4,40]. In Wang et al.’s study, a CNF-SO_3_Na-based TENG uses modified CNFs with improved triboelectricity or electricity storage properties to greatly increase electrical output [4]. The output performance of TENGs is a complex balance between charge density, dielectric doping effects and roughness events [40]. Adding fillers like ZnO or TiO_2_ can create a dispersion of nanocapacitors, reinforcing interface polarization and improving dielectric properties, as demonstrated in the study by Candido et al. [40]. Factors influencing electrical response include the specific surface area of the filler, nanoscale dispersion, filler content, onset of conductivity and matrix porosity [33]. Table 3 comparatively summarizes the principal surface engineering and dielectric modulation strategies employed in cellulose-based TENGs, highlighting the relationship between interfacial modification mechanisms, resulting structural effects, electrical behavior and associated processing or stability limitations.

The enhancement of cellulose-based TENGs usually relies on overcoming the biopolymer’s native weak tribo-polarity and low relative permittivity. As evidenced in the literature, the dominant mechanisms governing triboelectric enhancement involve a synergistic interplay between surface chemical engineering and internal dielectric modulation [1]. Modifying the cellulose main chain with highly polar or electronegative moieties (e.g., amination, fluorination) directly shifts the surface potential phase, regulating precise electron-donating or capturing capabilities [42]. More importantly, chemical interventions such as sulfonation extend beyond simple polarity shifts to engineer localized electrostatic deep traps, as noted by Wang et al. [4]. This molecular-level architecture noticeably suppresses the kinetic dissipation of triboelectric charges into the environment, thereby extending charge retention at the interface and bridging the performance gap with synthetic polymers [4].

Parallel to chemical functionalization, dielectric modulation via the integration of high-*k* or conductive nanofillers establishes dense microcapacitor networks that provoke intense interfacial polarization, significantly scaling up the equivalent capacitance [1,46]. However, these electrical enhancements introduce strict engineering trade-offs. Integrating inorganic or carbonaceous fillers frequently compromises the existing mechanical flexibility, optical transparency and ultimate biocompatibility/biodegradability of the cellulosic matrix [1,63]. Furthermore, filler loadings are rigidly bound by percolation thresholds because exceeding critical limits precipitates continuous electron tunneling and particular leakage currents that collapse TENG output [1]. From a physical engineering standpoint, while hierarchical micro-patterning maximizes the effective interfacial contact area, these delicate surface structures suffer from abrasive wear and microstructural fatigue during prolonged cyclic operation, deteriorating output stability [63]. Also, the scalable commercial implementation of these advanced cellulosic biointerfaces requires precise manufacturing control to balance ultrahigh triboelectric performance with environmental sustainability, mechanical durability and continuous processing stability.

Building upon the foundation of filler-induced interfacial polarization, the integration of nanofillers establishes a dense network of localized microcapacitors within the cellulosic matrix, which mainly alters the device’s equivalent capacitance (*C_TENG_*) and underlying internal impedance [1]. Mechanistically, optimized dielectric modulation scales *C_TENG_*, effectively decreasing the optimal matching impedance of the TENG by as much as 90% (e.g., from 2 MΩ to 200 kΩ) and triggering a coupled surge in maximum power output by a factor of 17.3, as highlighted by Du et al. [1]. However, this capacitive enhancement is strictly governed by the threshold for conductive network formation. Thus, filler concentrations that exceed this critical limit form contiguous electron tunneling pathways, which instigate considerable leakage currents, lead to electrical breakdown across the triboelectric interface and precipitate a sharp collapse in electrical output [1].

At the molecular scale, covalent surface engineering controls triboelectric dynamics by explicitly altering both the surface potential phase and native charge-trapping kinetics [4,69]. Because the density of transferred charges determines TENG electrical output, lowering the rapid kinetic dissipation of triboelectric charges into the environment remains relevant [4]. Introducing highly electronegative fluorine moieties via 1H,1H,2H,2H-perfluorodecyltriethoxysilane (PDOTES) silanization forces the surface potential of cellulose into a negative phase, thereby elevating its electron-capturing capacity and augmenting the surface charge density by 28.86%, according to Zhu et al. [69]. More profoundly, regulating the local electronic band structure through deep trap engineering serves to suppress charge dissipation entirely [4]. The substitution of hydroxyls with sulfonic acid (–SO_3_H) functional groups on cellulose nanofibers establishes a nearly symmetrical electrostatic potential distribution that functions as localized deep traps for holes [4]. This molecular modification expands the hole deep trap density by 714% and increases the dielectric constant by 116%, which effectively suppresses the dissipation rate of trapped charges into the surrounding environment by 77%, as noted by Wang et al. [4]. As a result, the sulfonated cellulosic matrix exhibits an ultrahigh triboelectric charge density of 92 μA/m^2^, representing a 460% amplification over pristine nanocellulose and demonstrating that molecular-scale deep trap engineering thoroughly connects microscopic polarization to macroscopic charge transfer efficiency [4].

### 3.4. Tribological Behavior and Triboelectric Performance of Cellulose-Based TENGs

The native hierarchical fibrillar organization of cellulose governs its bulk elasticity and topographical deformability, basically shaping the tribological contact mechanics during TENG operation [71,72,73,74,75,76,77]. The linear arrangement of cellulose chains, stabilized by hydrogen-bonding networks, affords particular mechanical resilience, allowing continuous cyclic deformation without structural fatigue [42,78,79,80,81,82]. By engineering the multiscale roughness and structural compressibility of the cellulose matrix, physical frictional interactions are optimized, facilitating superior contact electrification, efficient charge transfer and sustained triboelectric output [1,83,84,85,86].

Beyond internal capacitance, external contact mechanics are marked by the existing elastic modulus (E-modulus) and macroscale structural deformability of the cellulosic matrix. Modifying the thermodynamic regeneration pathways, such as utilizing longer-chain alcohols like *n*-pentanol instead of methanol, usually lowers the E-modulus of the resultant films [83]. This developed microstructural compliance leads to significant conformal deformation against soft counter-layers like polydimethylsiloxane (PDMS), noticeably amplifying the effective interfacial contact area to yield a >380% enhancement in output power [83]. By contrast, this compliance provides negligible advantage against highly rigid counter-layers like polytetrafluoroethylene (PTFE), where restricted topographically conformal contact limits charge generation [83]. In addition, macroscopic hierarchical deformability achieved through targeted mechanical creping induces specific spatial wavelengths and amplitudes in wrinkled cellulose architectures [45]. Under a dynamic mechanical stimulus, these considerably compressible matrices undergo significant volumetric deformation, which mechanically amplifies the relative capacitance and optimizes wave-driven contact-separation dynamics without the need for additional functional fillers [45].

Quantitative metrics reported in the literature include an output power of 120 mW·m^−2^ for a CMC-Na nanofiber film with a 3 × 3 cm area, as shown by Zhou et al. [12]; an output power density of 300 W·m^−2^ for cellulose and cellophane following dielectric modulation, reported by Du et al. [1]; and a maximum power density of 42 mW/m^−2^ for BC/ZnO nanocomposite films, as outlined by Jakmuangpak et al. [39].

## 4. Chitosan-Based Triboelectric Biopolymers

### 4.1. Processing–Structure Relationships in Chitosan-Based TENGs

Another biopolymeric material that was selected for the present study is chitosan (CS), which is the linear polysaccharide derived from the partial deacetylation of the most abundant component of the exoskeletons of crustaceans and insects, as well as the cell walls of fungi [87,88,89,90,91,92,93,94,95]. From the chemical perspective, the biopolymer is composed mainly of the units of D-glucosamine linked in the β-(1,4)-configuration, accompanied by some units of N-acetylglucosamine [87,95,96,97]. This biopolymer has gained considerable attention in the field of biomaterials for its native biocompatibility, biodegradability, low toxicity, low immunogenicity and various bioactivities such as antimicrobial and anticancer properties [6,9,87,88,96,97,98,99,100,101]. The biopolymer’s properties make it an important candidate for the development of environmentally friendly and biocompatible TENGs, as highlighted in various scientific studies [50,51,88,89,97]. The abundant hydroxyl and amino functionalities, together with the natural hydrogen-bonding capability of chitosan, contribute significantly to its film-forming ability, hydrogel formation and processability into structured triboelectric architectures [102].

Chitosan is described as biocompatible in the literature [2,6,9,10,11,39,50,51,52,53]. It has antimicrobial activity and good cytocompatibility, mentioned in Hidaka et al.’s study [9]. In addition, the interfacial behavior of biomaterials in aqueous environments, related to wettability and surface free energy, is significant for assessing biocompatibility [11].

**Figure 4:**
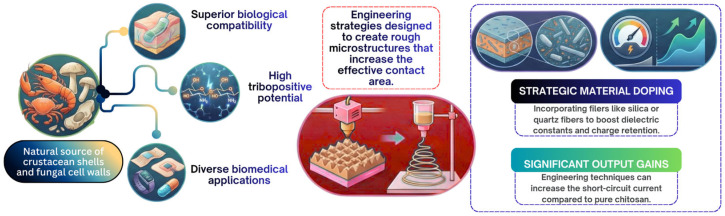
Main properties and engineering strategies that improve the triboelectric properties of chitosan-based materials.

Chitosan’s processability into various forms like films, fibers and hydrogels is important for TENG fabrication [5,9,103]. Solution casting is a common method for preparing chitosan films [50,51]. Also, electrospinning techniques can produce chitosan or chitosan-blend nanofibers [22,103]. Figure 4 shows the major material characteristics and performance-enhancing strategies related to chitosan-based TENGs.

Table 4 comparatively summarizes the principal processing-induced structural features reported in chitosan-based triboelectric systems, emphasizing the relationship between the fabrication route, resulting morphology, physicochemical behavior and their corresponding implications for triboelectric performance.

The triboelectric performance of chitosan-based systems is normally governed by processing-induced structural and morphological features [10,106]. Modulating the spatial architecture via electrospinning or phase-inversion generates highly porous, nanofibrous networks characterized by low compressive moduli [106]. This structural compliance maximizes the effective contact area and facilitates deep mechanical deformation under external stress, exponentially amplifying contact electrification [106,107]. Concurrently, embedding high-permittivity nanofillers (e.g., carbon nanotubes, clay nanosheets) or inducing ionic coordination within the biopolymer matrix drives macroscopic interfacial polarization and Maxwell–Wagner relaxation [104,106]. These compositional modifications establish deep charge-trapping sites that restrict electrostatic dissipation and substantially enhance the relative dielectric constant [104,113]. Nonetheless, engineering these frameworks involves strict trade-offs between porosity, flexibility and mechanical stability. While high porosity and liquid plasticization lower Young’s modulus to improve instantaneous contact dynamics, excessive void fractions or fluidic plasticizers may compromise the matrix’s tensile strength and structural integrity [89]. Likewise, overloading rigid nanofillers induces agglomeration, which embrittles the composite and obstructs continuous electron transport pathways [51,105]. A major limitation affecting scalability and long-term TENG operation is the hygroscopicity of pure chitosan. The moisture adsorption rapidly outperforms surface charges and collapses delicate micro-architectures [5,97,104], albeit chemical crosslinking and dual-network hydrogels can reduce these issues to confer anti-freezing or self-healing properties [111,112], ensuring the reproducible fabrication of these hierarchically custom architectures remains a particular challenge for implementing durable chitosan TENGs in ambient environments.

Chitosan presents several pivotal advantages for developing biomedical, implantable and environmentally responsive TENGs. Due to its biodegradability, biocompatibility and evident non-toxicity, it is optimal for applications involving contact with the human body, including skin-attachable [51,89,106,114] and wearable sensors [89,101,107,115], and potentially disposable medical products [89,109]. The antibacterial activity of chitosan is also highly beneficial for biomedical applications like wound dressings or implantable devices, potentially reducing the risk of infection [9,87,89,96,98]. Furthermore, chitosan-based TENGs can be designed to be fully degradable, reducing the environmental impact upon disposal [50,89,107,115].

As mentioned in the literature, some limitations are noticed, which are related to the fact that pure chitosan-based TENGs show lower output performance and mechanical properties compared to synthetic materials [5,88,99,115]. Chitosan films can also suffer from high brittleness [115] and low strength [96], necessitating strategies like plasticization or composite formation to improve flexibility and mechanical stability [89,115,116]. For tissue engineering applications, controlling pore size and ensuring consistent behavior with seeded cells can be challenging [6,96]. Additionally, chitosan derived from crustacean shells carries a potential risk of allergic reaction in humans, suggesting fungal sources as an alternative for certain biomedical applications [96]. Despite these limitations, ongoing research into material modification, composite formation and advanced processing techniques like AM continues to enhance performance and expand the potential applications of chitosan-based TENGs in the biomedical and sustainable energy sectors.

### 4.2. Additive Manufacturing Approaches for Chitosan-Based TENGs

AM can offer unique capabilities for creating structures with designed geometries and controlled surface properties [9,10,22,23,24,25], such as chitosan-based inks and hydrogels, which have been developed for extrusion-based 3D bioprinting [9,10,22]. By transitioning from stochastic to predictable structural control, 3D printing provides some advantages in customizing hierarchical porosity and structural anisotropy [117].

The rheological properties of chitosan formulations are mandatory for successful 3D printing and achieving the desired structural stability and shape retention [9,22,91]. A foundational requirement for extrusion-based printing is the shear-thinning (pseudoplastic) behavior of the hydrogel ink, wherein the apparent viscosity decreases significantly under the shear stress of the nozzle, facilitating smooth extrusion without clogging [22]. Upon exiting the micro-nozzle, the rapid recovery of the storage modulus over the loss modulus is required to ensure that the deposited filament maintains its structural integrity and resists gravity-induced collapse [22,118]. Nonetheless, researchers face a strict printability versus mechanical integrity trade-off [119]. While increasing the chitosan concentration or polymer molecular weight enhances the die swell ratio and post-printing shape fidelity, overly viscous inks can induce flow instability and restrict significant solvent diffusion [22].

For 3D bioprinting inks, improving gelation properties is a key challenge to ensure reliable printing and mechanical stability while maintaining biocompatibility [9]. Traditional post-printing gelation often relies on alkaline neutralization, where the protonated amine groups of chitosan are abruptly deprotonated [22,120]. Mechanistically, this rapid loss of electrostatic repulsion forces polymer chain packing and physical entanglement, which unfortunately manifests as serious macroscopic contraction and structural warping [22]. Such dimensional instability is a principal AM-specific limitation, as the evaporation of solvents and the neutralization process can reduce the filament volume significantly, resulting in wrinkled surfaces and substantial deviations from the intended engineered geometry [22].

To circumvent these rheological instabilities and structural collapse, advanced composite and dual-crosslinking strategies have emerged. Chitosan is an attractive material for developing inks for extrusion-based bioprinting of 3D structures due to its excellent properties [8,9]. Composite hydrogels based on chitosan and oxidized glucomannan can be formed through Schiff base and phenol crosslinking for extrusion-based bioprinting, as reported by Hidaka et al. [9]. Zhang et al. reported reinforcing chitosan hydrogels with silk particles for 3D printing [10], while Qin et al. employed laser surface texturing combined with mussel-inspired chitosan grafting to modify CoCrMo alloys [11]. Chitosan’s ability to form hydrogels makes it suitable for extrusion-based 3D bioprinting, allowing for complex 3D structures [9,10]. The dynamic equilibrium of the imine bonds in the Schiff base network grants the ink unique self-healing and stress-relaxing capabilities during extrusion, which are then permanently stabilized by secondary photo-induced crosslinking [9]. Also, the inclusion of rigid nanofillers dramatically increases the compressive modulus and mitigates drying-induced shrinkage by acting as physical barriers that restrict polymer chain mobility [10,87]. Table 5 comparatively summarizes the principal AM strategies employed for chitosan-based TENG architectures, highlighting the relationship between printable formulation design, rheological behavior, resulting structural organization and their corresponding triboelectric implications.

An analysis of AM strategies for chitosan-based TENGs reveals a distinct processing-induced trade-off between structural resolution, mechanical resilience and triboelectric performance. Extrusion-based DIW and hybrid composite printing offer good scalability and the ability to algorithmically integrate high-permittivity dielectric fillers (e.g., quartz fibers), which directly establish interfacial polarization centers to enhance charge retention and macroscopic power density. Notwithstanding this, these techniques frequently battle particular drying-induced contraction, rheological instabilities and nozzle clogging when matrix viscosity is increased to preserve shape fidelity. Contrariwise, electrospinning sidesteps conventional macroscopic printing limitations by producing very porous, nanofibrous networks with considerably low compressive moduli, significantly maximizing the instantaneous contact area and electrostatic induction during cyclic mechanical deformation. Even as advanced methods such as DLP lead to distinctive geometric complexity and refined micro-architectures, their considerable reliance on aqueous environments and limited photo-crosslinking substitution degrees innately suppress triboelectric charge accumulation without rigorous post-processing desiccation. Thus, the advancement of stable chitosan TENGs relies on hybridizing these AM techniques, such as combining micro-patterned templating with dual-plasticized or filler-reinforced inks, to integrate macroscale deformability with nanoscale charge trapping, effectively overcoming the performance limitations of pure biopolymers in self-powered sensing environments.

Essentially, the implementation of AM significantly changes the microstructural and mechanical landscape of chitosan frameworks. By algorithmically controlling the deposition paths, 3D printing imparts defined anisotropic properties and interconnected porosity that cannot be replicated by traditional casting [22,117]. These engineered architectures directly translate to enhanced mechanical resilience and fatigue resistance under cyclic deformation [117]. By alleviating structural collapse and enabling high-resolution geometric fidelity, AM ensures that the resulting matrices can sustain requisite physical stresses, maximizing the reliable contact area and long-term durability of the functional architecture [10,117].

### 4.3. Surface Engineering and Dielectric Modulation of Chitosan-Based TENGs

Controlling surface roughness and porosity is essential for optimizing the triboelectric performance of biocompatible materials like chitosan for applications in TENGs and AM systems. While pristine chitosan films are relatively smooth, with a root-mean-square (*R_a_*) roughness around 3.38 nm [51], various methods can engineer its surface texture and porosity. The surface morphology of chitosan films can vary depending on additives [8]. Adding activated carbon (AC) and sodium chloride (NaCl) can increase the surface roughness of chitosan films [8]. Pristine CS films can have a smooth surface texture, while the addition of additives like graphene nanoplatelets (GNPs) results in a rough morphology, as mentioned by Maity et al. [5]. Blending chitosan with different polycations can produce distinct nanoscaled surface topographical features, such as particles, granules, fibers and islands [6]. Moreover, chitosan grafting can increase the wetting performance (decrease the contact angle) of surfaces, indicating high hydration [11].

Techniques to modify chitosan surfaces include blending with inorganic fillers such as clays (sepiolite, bentonite, kaolin) which create coarser surfaces with visible valleys [51] or AC and NaCl, significantly increasing roughness up to 770.98 nm [8]. Incorporating plasticizers like glycerol and polyethylene glycol also improves surface roughness, as reported by Gao et al. [89]. Electrospinning can create rough nanofiber membranes [89,103]. Electrospun nanofiber membranes can exhibit a rough structure [107], which is beneficial for enhancing triboelectric performance by increasing the effective contact area. Other strategies involve replicating microstructures like sandpaper or constructing porous architectures (Han et al.) [108], embedding particles such as quartz fibers or SiO_2_ nanoparticles (Liu et al.) [115], metal-ion coordination (e.g., Mg^2+^) which increases roughness by 39.4 nm (He et al.) [99], ion etching which can increase RMS roughness (Ko et al.) [122] or blending with polycations to yield various nanoscale topographies (Zheng et al.) [6]. Its surface properties, including nanotopography, chemistry and wettability, can be tuned through blending or grafting, influencing cell behavior, as mentioned in Zheng et al.’s study [6]. The surface topography of chitosan materials may play an important role in regulating nerve cell behavior, suggesting that topographic modification can be used for tissue regeneration applications [6]. For porosity control, methods like emulsion freeze-drying combined with directional freezing can produce porous scaffolds with controllable pore diameters ranging from 40 to 112 μm (Ezeldeen et al.) [96], which is relevant for AM of structured materials.

These surface modifications directly impact triboelectric behavior. An enhancement in surface roughness is believed to be useful for enhancing TENG performance as it increases the contact area, thereby improving friction and charge generation [8,40]. Additives and modifications can increase the surface potential (e.g., from 3.64 mV for pure CS to 22.92 mV for CS/AC/NaCl), as reported by Chaturvedi et al. [8], and enhance dielectric properties by forming polarization centers, boosting charge retention and transfer capabilities [99,115]. The addition of AC, reported by Chaturvedi et al., increased the surface potential of the CS-AC layer, and further insertion of NaCl enhanced it, potentially due to improved electron-donating ability [8]. In addition, chitosan brushes on a textured surface can contribute to reducing friction force and promoting the formation of a local lubricating film [11]. Also, tailoring ionic salt content in chitosan-activated carbon composites can influence triboelectric performance [8].

Consequently, numerous strategies have been developed to enhance the triboelectric output performance of chitosan, including doping, surface treatment, ion embedding and structure engineering, as emphasized by Fang et al. [97]. These methods often focus on modulating the surface properties, dielectric constant and charge transfer capabilities of the chitosan material. Surface modification techniques, such as molecular surface engineering, have been explored to modify functional groups and significantly boost the performance of chitosan-based TENGs [88,89]. Incorporating various fillers into chitosan composites is a common approach to enhance properties. For instance, adding organic proteins [89,101,103,109] or inorganic fillers like clay (sepiolite, bentonite, kaolin) [51,89], activated carbon [8] or inorganic oxide particles such as BaTiO_3_, TiN, SiO_2_ or quartz fiber [8,99,106,113,115] can improve performance. Embedding oxide particles, including SiO_2_, has been shown to create polarization centers within the composite, thereby boosting the material’s dielectric constant and enhancing charge retention capacity [8,115]. Regulating the surface microstructure and dielectric constant is a key strategy for preparing high-performance chitosan-based TENGs [5,106,109,110,123].

Table 6 comparatively summarizes the principal surface engineering and dielectric modulation strategies employed in chitosan-based TENGs, emphasizing the relationship between interfacial modification mechanisms, resulting structural effects, electrical behavior and the associated processing or stability limitations.

A comparative analysis of surface-engineering strategies in chitosan-based TENGs reveals that the natural triboelectric performance of pure chitosan is fundamentally limited by its dense hydrogen-bonding network, which restricts dipole mobility and facilitates rapid charge dissipation. To circumvent this, strategies such as dielectric nanofiller incorporation (e.g., carbon nanotubes, Ag nanowires) and ionic modulation (e.g., Mg^2+^ or Ca^2+^ coordination) are usually employed to induce Maxwell–Wagner interfacial polarization and establish particular charge-trapping sites, thereby exponentially increasing the relative dielectric constant and surface potential. Also, chemical functionalization, such as tannic acid grafting or quaternization, directly dismantles restrictive intra-chain hydrogen bonds, effectively liberating lone electron pairs and providing the unique capability to completely reverse or dramatically magnify the material’s triboelectric polarity. Mechanically, micro- or nanostructuring via electrospinning or templating considerably lowers the compressive modulus and expands the effective contact area, maximizing electrostatic induction under dynamic stress. Nonetheless, these optimizations present careful engineering trade-offs. Although extreme porosity and hygroscopic ionic doping enhance instantaneous contact mechanics and charge density, they simultaneously expose the biopolymer to particular moisture sensitivity, structural collapse and charge screening (leakage currents) in ambient environments. Meanwhile, excessive loading of rigid nanofillers to boost permittivity inevitably triggers agglomeration and mechanical embrittlement, highlighting the necessity for hybridized processing routes that integrate nanoscale dielectric modulation with macroscale structural integrity to achieve durable, high-performance biopolymeric TENGs.

These improvements translate to enhanced TENG outputs, with examples reaching open-circuit voltages up to 173 V (Gao et al.) [89], short-circuit currents up to 20.2 μA (Chaturvedi et al.) [8] and power densities of 37.8 mW/m^2^ (Liu et al.) [115]. Conversely, the degree of dispersion of fillers like GNPs in the chitosan matrix affects performance; aggregation can negatively impact the performance of the triboelectric material [5]. In the study by Chen et al., maintaining a dense contact surface through strong intermolecular interaction in composites can help restrain the volatilization of induced electrons, which is important for performance, particularly at higher temperatures [95].

Chitosan’s natural biocompatibility and biodegradability make surface-engineered versions advantageous for wearable or implantable TENGs [5,88,97,99] and its processability allows for integration into AM systems [9,25,87]. However, pure chitosan’s naturally low surface charge density and mechanical limitations necessitate these modifications to achieve competitive performance [99]. Therefore, tailoring surface texture and porosity is an important approach for developing high-performance, biocompatible and potentially AM chitosan-based TENGs.

### 4.4. Tribological Behavior and Triboelectric Performance of Chitosan-Based TENGs

The biopolymer was traditionally applied as the tribopositive material in the field of TENGs [51,89,97,99,116]. The triboelectric properties of the biopolymer are attributed mainly to the large number of primary hydroxyl (–OH) and secondary amino (–NH_2_) functional groups present in the backbone of the biopolymer, acting as electron donors [5,89,97,99]. The chitosan-based TENGs are promising for the development of wearable electronics [89,91,115], electronic skin [89,107,115], self-powered sensing technology [9,50,89,95,107,115,124,125,126,127,128,129] and medical disposables [89,109,123,130].

During contact electrification with highly electronegative counterpart materials (such as PTFE or FEP), the lone electron pairs positioned on the nitrogen and oxygen atoms of chitosan transfer across the interface, leaving the chitosan surface positively charged [5,105]. Nonetheless, the dense intra- and intermolecular hydrogen-bonding network within pure chitosan seriously restricts the mobility of its polarizable groups, often resulting in comparatively weak electron donation, low surface charge density and rapid charge dissipation [8,110]. To overcome these particular limitations, researchers have focused on basically altering the synthesis–structure–property relationships of chitosan matrices by engineering the dielectric behavior, ionic mobility and surface structural dynamics.

A common approach to enhance triboelectric output relies on dielectric modulation and interfacial polarization. Incorporating high-permittivity nanofillers induces Maxwell–Wagner relaxation at the filler–matrix interface, creating deep charge-trapping sites that effectively restrict the annihilation and dissipation of triboelectric charges [104,106]. For instance, embedding MWCNTs at an optimal 0.8 wt% into a chitosan matrix increased the dielectric constant by several orders of magnitude, amplifying the open-circuit voltage to 85.8 V and delivering a power density of 180 mW/m^2^, which represents a staggering 82-fold and 5-fold enhancement in voltage and transferred charge, respectively, over pure chitosan (Han et al.) [106]. Inorganic nano-clays behave similarly. Doping chitosan with biocompatible bentonite clay (1 wt%) considerably elevated the surface potential and contact electrification, generating an outstanding 996 V and a peak power density of 26.5 W/m^2^ (Yar et al.) [51].

Outside physical fillers, chemical modification of functional groups can significantly shift the electron transfer mechanism by dismantling the restrictive hydrogen-bonding networks. Synthesizing single-phase chitosan derivatives, such as chitosan quaternary ammonium salt (CQAS), physically unbinds the lone electron pairs on O and N atoms, elevating the relative dielectric constant by 37 times and maximizing the transferred charge density to 3.0 nC/cm^2^ (Zheng et al.) [110]. Remarkably, triboelectric polarity is not an immutable property. Some specific chemical modifications can completely reverse it. The surface grafting of tannic acid onto chitosan introduces galloyl groups and aromatic rings that engage the native –NH_2_ electron-donating sites and this structurally shifts the overall surface electron affinity, efficiently reversing the material’s triboelectric polarity from positive to relatively negative while achieving a stable charge density of 182 μC m^−2^ (Fang et al.) [97].

The intentional induction of ionic polarization through salt coordination represents another particular leap in performance optimization. For example, in the study by Liu et al., embedding quartz fibers in chitosan led to a CQ-TENG with a time-averaged power density of 37.8 mW/m^2^, which was 3.3 times greater than a pure chitosan TENG [115]. The inorganic quartz fibers act as stable space charge polarization centers through electrostatic interactions, thereby fortifying the charge retention capacity of the positive triboelectric layer [115]. Also, in the study by Chaturvedi et al., the addition of activated carbon and ionic salt (NaCl) to chitosan composites also resulted in significant improvements in output voltage (boosted by 2.55 times) and short-circuit current (boosted by 15.67 times) [8]. From a mechanistic standpoint, the integration of free ions (such as Na^+^, Ca^2+^ or Mg^2+^) establishes coordinate bonds with the hydroxyl and amino groups of chitosan [99,113]. This kind of coordination interrupts dense hydrogen bonding, sharply increases dipole density and establishes channels for superior ionic mobility, which in the end amplifies the saturated surface charge density during contact-separation cycles [8,99].

Eventually, modifications aimed at surface roughness and porosity exponentially expand the effective interfacial contact area, driving macroscopic friction enhancements. Gao et al. have shown that techniques like dual plasticizing with glycerol and polyethylene glycol improve surface roughness and introduce additional –OH groups, leading to enhanced tribopositive electrical generation and a significant increase in open-circuit voltage (up to 173 V, three times higher than pure CS-based TENGs) [89]. Corresponding architectural benefits are realized by embedding highly porous diatom bio-silica into chitosan, wherein the distinctive nanoporosity and hydrogen-bonding interactions with silica maximize the electro-positivity of the film, delivering a 3.7-fold increase in power density (15.7 mW/m^2^) compared to planar films [105]. Likewise, forming porous chitosan networks via electrospinning results in a low compressive modulus that permits more significant structural deformation under external forces, contributing to a marked increase in the frictional contact area and yielding superior current and voltage outputs compared to inherently smooth, casted films [108]. By synergizing these dielectric, ionic, and morphological optimizations, chitosan-based frameworks shift from functioning as weakly performing natural biopolymers to high-output, highly responsive triboelectric matrices.

## 5. Silk Fibroin-Based Triboelectric Biopolymers

### 5.1. Processing–Structure Relationships in Silk Fibroin-Based TENGs

Silk fibroin (SF), primarily sourced from the cocoons of the domesticated silkworm Bombyx mori, stands out as a highly promising natural biopolymer for applications in bioelectronics and energy harvesting, particularly within the domain of TENGs [2,131,132,133,134,135,136,137,138,139,140]. Comprising ~70–80% of the silkworm silk fiber, SF is typically isolated after the removal of sericin, a glue-like protein, through a process called degumming [131,135,141,142,143,144,145,146,147,148]. This purification is important as residual sericin can potentially cause compatibility issues and allergic reactions, as mentioned in Xu et al.’s study [144].

In 1993, the U.S. Food and Drug Administration classified silk fibroin as a material with good biocompatibility and biodegradability [131,135,136,141,142,145,146,147,148,149,150]. In addition, silk fibroin also exhibits good processability and favorable mechanical properties, which further supported its classification as a safe material for use. Silk fibroin is basically a protein composed of a light chain with a molecular mass of approximately 25–26 kDa and a heavier chain with a molecular mass predominantly ranging between 325 and 390 kDa [131,134,141,142,147]. The heavy chain consists of repetitive Gly–Ala–Gly–Ala–Gly–Ser sequences that tend to form stable antiparallel β-sheet crystallites, which are responsible for the well-known stiffness and strength of this material [131,141,142,151].

These crystalline regions are interspersed with non-repetitive amorphous regions composed of amino acids with larger side chains [142,151]. As a result, SF can exist in different structural forms, notably Silk I (a metastable form with β-turns and α-helices) and the more stable Silk II (a β-sheet structure), with the transition from Silk I to Silk II often induced by treatments like methanol or potassium phosphate [131,142,151]. Nettey-Oppong et al. emphasize that the structural conformation directly impacts the material’s properties, including its mechanical and optical characteristics [151]. Thus, processing-induced modulation of the Silk I/Silk II ratio directly governs the mechanical compliance, structural stability and tribological behavior of SF-based TENG layers.

Table 7 comparatively summarizes the principal processing-induced structural features reported in SF-based triboelectric systems, highlighting the relationship between the fabrication route, resulting morphology, physicochemical characteristics and their corresponding implications for triboelectric performance.

The optimization of SF-based TENGs basically relies on customizing processing–structure–property relationships to maximize contact electrification and mechanical durability [156,157,158,159,160]. Dominant processing strategies have transitioned from conventional solvent casting of dense 2D films to advanced additive and assembly techniques, such as electrospinning, directional freeze-drying and electrospray-etching, which engineer hierarchical 3D architectures [7,153,155]. Morphologically, transitioning from planar films to interconnected nanofibrous or aerogel networks considerably amplifies the specific surface area and volumetric compressibility, directly enhancing the effective tribological contact area and facilitating internal charge trapping within porous matrices [7]. Also, controlling the β-sheet crystallinity through alcohol annealing or vapor treatment is consequential. Although it bolsters mechanical stability and cyclic durability against abrasive wear, excessive crystallinity can compromise the elastomeric flexibility necessary for conformal contact [152,157].

Dielectric and structural engineering through the incorporation of dopants (e.g., Li^+^ ions, MXene, CNTs) jointly modulates interfacial and ionic polarization, augmenting the natural electron-donating capacity of the SF amide groups and minimizing internal resistance [17,156]. Despite these notable improvements in power density, significant engineering compromises persist. For instance, while hygroscopic ionic salts or highly polar fillers elevate dielectric constants, they exacerbate moisture sensitivity, leading to rapid charge dissipation and performance degradation in high-humidity environments [156]. Moreover, high loading fractions of conductive nanofillers often result in agglomeration, compromising both the structural integrity and electrical uniformity of the composite matrix [17,136]. Eventually, the realization of scalable and durable SF-TENGs requires resolving these trade-offs by developing hybrid manufacturing frameworks that ensure uniform filler dispersion, long-term phase stability under cyclic mechanical stress and resilient encapsulation strategies to isolate the active triboelectric interfaces from environmental humidity [136,156].

**Figure 5:**
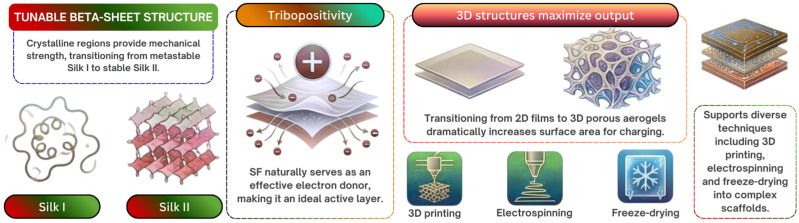
Main structural, physicochemical and fabrication-related attributes of silk fibroin relevant to TENG performance.

An integrated overview of the main structural, physicochemical and manufacturing features that govern the triboelectric performance of silk fibroin-based systems is presented in Figure 5.

SF is known for its excellent biocompatibility and bioabsorbability, making it suitable for implantable electronics [2,3,7]. SF-based TENGs can be skin-friendly and biodegradable, as reported by Su et al. [138]. While surface engineering is used to boost performance, maintaining inherent biocompatibility is mandatory, especially for medical or wearable applications [2,3,7]. Highly porous, ultralight structures can be achieved, which are advantageous for wearable devices, while also maintaining mechanical stability and biocompatibility [7].

The primary trade-off discussed is between enhancing triboelectric performance (often requiring surface modifications, fillers or structural changes) and maintaining the inherent biocompatibility, biodegradability and desired mechanical properties (flexibility, strength) necessary for wearable or implantable devices [1,4]. For instance, while adding certain fillers or performing specific chemical modifications can increase charge density or surface area, they might alter the material’s mechanical integrity or biological interactions [4,5,6]. Similarly, processing into highly porous structures can increase surface area and output but might affect mechanical robustness [3]. The goal is to achieve a balance where performance is optimized without sacrificing important biocompatibility and mechanical characteristics [4,7].

The most significant benefits of SF for implantable, resorbable and wearable triboelectric applications lie in its natural biocompatibility, biodegradability and non-immunogenicity, making it suitable for direct contact with the body [131,135,136,141,142,145,146,147,148,158]. Moreover, its flexibility allows for integration into wearable devices [132,136,159]. The tunable degradation rate is beneficial for temporary implants or resorbable electronics [131,135,144,147]. SF-TENGs offer the potential for self-powered wearable sensing and biomedical devices [2,3,7,159]. Nevertheless, challenges remain, including the need to enhance the mechanical strength of SF constructs for applications requiring load-bearing capability [135] (though less critical for typical TENG layers), precisely controlling the degradation rate in vivo [135,147], and ensuring optimal integration with other materials in complex systems [135,147]. Notwithstanding these challenges, the ability to tailor SF’s properties and structure through advanced fabrication methods, including AM, positions it as an important material for developing next-generation biocompatible and potentially resorbable TENGs and self-powered biomedical devices.

### 5.2. Additive Manufacturing Approaches for Silk Fibroin-Based TENGs

Standard fabrication approaches for SF-TENGs, such as spin coating, solvent casting and spray coating, usually constrain the structural complexity of the resulting devices to planar, two-dimensional architectures [132,160]. While these basic methods offer simplicity, they struggle to provide the precise multiscale spatial control required to optimize triboelectric surface area and mechanical compliance [161]. In stark contrast, AM approaches, including DIW and stereolithography, facilitate the bottom-up, programmable assembly of complex 3D structures, representing a strategic transition in SF-based device fabrication [162]. Nonetheless, native regenerated silk fibroin (RSF) solutions present considerable rheological challenges for AM due to their low viscosity and poor shape fidelity [163]. To successfully transition from conventional casting to 3D printing, SF inks must be formulated to exhibit shear-thinning behavior, a defined yield stress and rapid post-extrusion viscosity recovery [162]. This is commonly achieved by functionalizing the SF structural basis, such as through methacrylation (Sil-MA) or norbornene modification to enable rapid photo-crosslinking, or by introducing rheological modifiers like Konjac gum and alginate [162,164,165]. These rheological interventions largely govern the printability of the SF inks, directly determining the structural resolution and subsequent triboelectric efficiency of the fabricated TENGs [166].

SF’s excellent processability allows for its fabrication into various forms relevant to triboelectric applications and AM techniques [131,135]. Regenerated SF solutions, obtained through steps including degumming, dissolution in salt solutions (like LiBr) and dialysis [131,132,143,163], serve as the base material. As reported in the study by Qi et al., these solutions can be processed into particles, fibers, films, hydrogels and sponges [131]. AM and printing techniques offer precise control over the creation of structured SF materials. Electrospinning is a widely employed fiber fabrication technique to produce SF nanofibers, which can be incorporated into TENGs or used to create textured surfaces [145,146,148,154,159,162,167]. Findings by Choi et al. indicate that spin coating is employed to produce thin SF films with controlled thickness (ranging from 81.9 nm to 264.8 nm) on various substrates, impacting the optical, electrical and dielectric properties, including capacitance and leakage current [132]. 3D printing techniques, such as extrusion-based printing and direct-write assembly, enable the fabrication of hierarchical or microperiodic SF scaffolds and structures [131,162,163]. Also, freeze-drying is used to prepare porous aerogels and sponges from SF solutions [3,136,144], while novel methods like gas-foaming can expand 2D SF fibers into 3D microfiber scaffolds [144].

Most methods include creating highly porous silk aerogels from silk fibroins extracted from cocoons [3,7]. Electrospinning has been used to develop one of the first silk-fiber-based TENGs, enabling the fabrication of highly porous and nanoscale fiber networks that significantly enhance surface area and promote efficient charge generation [2,3,13,14,15,16]. Su et al. reported that films can be produced from SF or by electrospraying a highly conductive mixed solution onto a silk-fiber substrate [138]. Other methods describe water electrospray-etching, which can be used to create porous and hierarchically structured silk fibroin films [2]. Electrospinning yields nanofibers, while aerogel fabrication often involves freeze-drying to preserve porosity. Table 8 summarizes the prominent AM and biofabrication strategies employed to engineer hierarchical silk fibroin architectures for advanced TENGs.

The integration of AM into the fabrication of SF-based TENGs marks an important shift from planar casting to structurally engineered 3D architectures [162,163]. Even though template-assisted soft imprinting effectively imparts surface roughness to maximize interfacial contact, it remains constrained by multi-step processing and dimensional contraction during post-fabrication β-sheet crystallization [157,160]. Instead, DIW and extrusion bioprinting offer notable volumetric control, leveraging shear-thinning and rapid viscoelastic recovery to construct highly porous, load-bearing matrices that amplify compressive deformability and internal charge trapping [162,163]. However, the rheological requirements for DIW frequently involve considerable high SF concentrations or the integration of rheological modifiers, which can precipitate premature nozzle clogging and complicate post-processing solvent extraction [162]. To avoid these extrusion bottlenecks, DLP utilizing methacrylated SF (Sil-MA) achieves superior spatial resolution and structural fidelity through rapid photo-crosslinking [168,170]. DLP facilitates the formation of dense, dual-network composite hydrogels (e.g., doped with PEDOT:PSS) that particularly modulate dielectric properties and interfacial polarization, though curing depth limitations and filler agglomeration remain important engineering challenges [169]. Concomitantly, hybrid approaches combining electrospinning and electrospraying sidestep bulk macro-architecting to focus on the nanoscale, generating in situ interlocked, highly flexible nanofibrous networks via co-solvent anchoring [171]. These electrohydrodynamic methods coordinate contact mechanics. The resulting remarkably high surface-to-volume ratio exponentially boosts contact electrification, while the co-spraying of fluorinated nano-electrets ensures durable charge retention within the highly permeable matrix [171]. Likewise, ESE exploits electrostatic induction to dissolve localized SF domains, creating quasi-periodic hierarchical porosity that clearly enhances surface charge density [155]. Thus, the positive scaling of these AM strategies relies on overcoming inherent processing concessions, such as the balance between β-sheet-induced wear resistance, structural brittleness and hygroscopic instability, to ensure the reproducible fabrication of efficient, mechanically compliant SF-TENGs [155,163,169].

The defining advantage of AM in SF-TENG fabrication resides in the direct translation of programmed fabrication parameters into optimized microstructural hierarchies, establishing a deep synthesis–structure–property relationship. 3D printing combined with sacrificial templating, or the use of directional freeze-drying to create ultralight aerogels, allows for the accurate production of tunable, interconnected macropores [7,162]. Therefore, these hierarchical architectures significantly change the triboelectric charge dynamics. Instead of merely accumulating at the planar interface, triboelectrically induced charges diffuse and reside securely on the vast interior pore surfaces [17].

Beyond extrusion-based 3D printing, the collaborative integration of electrospinning and electrospraying provides a resilient AM strategy to modulate surface roughness and tribological mechanics at the nanoscale [154,159]. To deal with this, the synchronous electrospinning of SF and electrospraying of functional nanoparticles (e.g., fluorinated SiO_2_) can generate in situ interlocked, flexible microfibers via a co-solvent anchoring effect, circumventing the need for exogenous chemical binders and resulting in highly resilient, permeable TENG layers [171]. Alternatively, a new ESE technique utilizes atomized droplets to simultaneously dissolve water-soluble SF domains and deposit residual charges, creating a localized, micro-patterned electric field [155]. The superposition of these electric field forces orchestrates the formation of quasi-periodically distributed nanopores, effectively integrating nano-electrets into the hierarchical matrix [155]. This structurally etched topography markedly improves the interfacial roughness, boosting the triboelectric output by 2.6 times compared to smooth planar films while preserving excellent long-term cyclic stability over thousands of deformation cycles, as reported by Luo et al. [155]. In addition, controlling processing parameters like film thickness in techniques such as spin coating is vital, as it affects electrical properties like leakage current and dielectric loss [132].

To further maximize triboelectric output, AM processes are significantly paired with composite ink engineering to achieve targeted dielectric modulation of the SF matrix [156]. The insertion of 2D nanomaterials such as MXene, CNTs, or the doping of metal cations (e.g., Li^+^, Histidine) into the printable SF dope particularly enhances both ionic and interfacial polarization [138,156]. For instance, formulating an SF composite ink with 40 wt% MXene enriches the surface with –OH and –F functional groups, substantially elevating the surface charge density and pushing power densities up to 9.92 W/m^2^ (Tan et al.), a 3.8-fold improvement over neat SF configurations [172]. Despite these impressive structure–property enhancements, scalability and manufacturing limitations remain as central difficulties [159]. Although techniques like coaxial 3D printing, resulting in conductive carbon-core/SF-sheath fibers, demonstrate a pathway toward scalable, highly integrated TENG textiles, the uniform dispersion of conductive nanofillers and the long-term phase stability of these composite bio-inks under high shear stresses remain engineering challenges that must be addressed to transition from laboratory prototyping to commercial mass manufacturing [159].

### 5.3. Surface Engineering and Dielectric Modulation of Silk Fibroin-Based TENGs

Silk fibroin is a highly versatile biomaterial, extensively processed into various forms for biomedical applications, including those requiring controllable surface texture and porosity [131]. This structural control is particularly relevant for developing biocompatible TENGs, as mentioned by Gan et al. [2]. Some techniques enable tuning SF surface morphology and porosity. Kim et al. described how electrospinning yields highly porous nanofiber membranes, such as those with an average diameter of 80 nm and 76.1% porosity [145]. Also, Choi et al. outlined how spin coating produces thin films (e.g., 81.9 nm to 264.8 nm thick) with consistent surface roughness (around 1.5 nm) on smooth substrates, important for dielectric properties in bioelectronics [132]. Thus, AM via 3D printing allows the generation of structures with controlled porosity and hierarchical features also in the case of SF [131,162,167]. Porous scaffolds can also be fabricated using methods like salt leaching, employing materials like NaCl or polymer particles to template pore formation, as noted by Wen et al. [159]. Xu et al. showed that gas-foaming techniques can expand 2D SF fibers into 3D porous structures [144]. Furthermore, SF aerogels, prepared through freeze-drying techniques, exhibit high porosity and nanofibrillated structures, with surface areas tunable by concentration (e.g., 18.5 m^2^/g for 2% SF aerogel, highlighted by Mi et al.) [3].

Silk aerogels can exhibit a nanofibrillated porous structure and high surface area, depending on the concentration of the solution used [3,7]. In Mi et al.’s study, raw silk fibers show a nonwoven structure with an average diameter of around 6 μm [3]. Therefore, CNF/silk aerogels can exhibit a complex surface structure including a nanofibrillated structure and film- or ribbon-like structures [3]. The porosity and surface area of silk aerogels can vary. For example, a 1% solution silk aerogel had lower porosity and surface area than a CNF aerogel, as shown by Mi et al. [3]. Tan et al. highlights how transitioning from 2D film friction layers to 3D porous aerogels significantly increases the specific surface area [7].

The surface texture and internal porosity significantly influence triboelectric performance by increasing the effective contact area for charge generation [3]. Highly porous structures and rough surfaces facilitate this process [3]. Notably, SF aerogel-based TENGs leverage their high surface area from nano-structured surfaces and internal pores to enhance output [3,7]. The nanofibrillated porous structure and higher surface area of certain silk aerogels contribute to high triboelectric output performance [3,17,18,19,20,21]. Increased surface area provides more area for charge generation, both on the external surface and the surfaces of the inner pores [3]. Compared to SF film-based TENGs, SF aerogel TENGs have demonstrated substantial performance improvements, including a 6.5-fold increase in voltage and a 4.5-fold increase in current, achieving outputs up to 365 V and 11.8 µA for a 3% SF aerogel, as demonstrated by Tan et al. [7].

Additional studies further demonstrate that porous and hierarchically structured SF films can be fabricated to enhance energy harvesting [7]. Also, modifying SF films by embedding in-plane aligned nanoflakes can significantly enhance the output performance of TENGs [2,144,160]. Thinner spin-coated films also affect electrical properties like leakage current and resistance due to shorter charge paths, as noted by Choi et al. [132]. Besides morphological optimization, dielectric modulation in SF-based TENGs carries an important role in regulating charge generation, retention and transfer dynamics. The insertion of functional nanofillers, conductive nanoflakes or polar dopants into the SF matrix brings localized interfacial polarization sites that enhance dielectric permittivity and suppress rapid charge dissipation. These dielectric modifiers facilitate dipolar and interfacial polarization mechanisms, thereby increasing surface charge density and improving charge retention during repeated contact-separation cycles. Concomitantly, hierarchical porous architectures generated through freeze-drying, electrospinning or AM increase the density of accessible charge-trapping regions within the internal structure of the material. These effects create a coupled structure–dielectric relationship in which surface roughness, pore topology and interfacial polarization together govern triboelectric effectiveness. Nonetheless, excessive filler loading or uncontrolled pore enlargement may compromise mechanical stability, increase leakage current pathways and reduce long-term cyclic durability, revealing that optimization of SF-based triboelectric systems requires balancing dielectric enhancement with structural integrity and mechanical compliance. Table 9 comparatively summarizes the principal surface engineering and dielectric modulation strategies employed in SF-based TENGs, emphasizing the relationship between interfacial modification mechanisms, resulting structural effects, electrical behavior and the associated processing or stability limitations.

The assessment of the strategies summarized in Table 9 reveals that the triboelectric performance of SF-based systems is usually governed by the interplay between dielectric modulation, interfacial polarization and hierarchical surface structuring. Approaches such as MXene incorporation, ionic doping and CNT integration considerably strengthen charge density and electrical output by improving dielectric properties and charge transport pathways. Meanwhile, electrospinning and electrospray-etching increase the effective contact area through porous and roughened micro/nanostructures. Nonetheless, these enhancements are frequently associated with important concessions, including humidity-induced charge dissipation, filler agglomeration, structural brittleness and reduced long-term mechanical stability. Because of this, achieving durable high-performance SF-based TENGs requires a balanced integration of dielectric engineering and structural optimization while maintaining environmental and mechanical stability.

These surface-engineered SF materials, combined with SF’s natural biocompatibility, are promising for wearable bioelectronic devices and self-powered sensors [2,7]. Despite these advances, difficulties associated with AM processing remain, including achieving suitable ink rheology for high-resolution printing, maintaining uniform pore architectures and preventing nanofiller aggregation during fabrication [163,167]. These kinds of limitations may detrimentally affect dielectric homogeneity, mechanical durability and long-term triboelectric stability, highlighting the need for further optimization of SF-based manufacturing strategies for scalable and reliable TENG fabrication.

### 5.4. Tribological Behavior and Triboelectric Performance of Silk Fibroin-Based TENGs

The tribological behavior and succeeding charge transfer efficiency of SF-based TENGs are mainly determined by the biphasic molecular architecture of the protein. The rigid β-sheet nanocrystals, functioning as physical crosslinks, impart substantial mechanical strength, stiffness and friction resistance, which are critical for enduring cyclic contact-separation without harsh wear or structural fatigue [176,177]. Contrarily, the amorphous domains (comprising random coils and α-helices) provide the requisite elastomeric flexibility, facilitating the friction layer to achieve intimate conformal contact with counter-surfaces under mechanical loading [156,178]. This structural integration determines the friction-induced charge transfer mechanism; as the SF layer deforms and conforms to a tribonegative surface, its abundant electron-donating functional groups are brought into optimal proximity for interfacial electron transfer, thereby maximizing contact electrification [7,157,179]. Also, processing strategies that modulate the crystalline-to-amorphous ratio, such as alcohol or water vapor annealing, directly influence the material’s yield stress and elastic recovery, optimizing the balance efficiently between structural durability and effective tribological contact area during repeated mechanical loading [155,178,180].

Amine groups present in silk fibroin are electron-donating groups that tend to lose electrons, which can enhance triboelectric performance (Mi et al.) [3]. Therefore, the triboelectric output performance can be adjusted by varying the ratio of silk in hybrid aerogels [3]. Quantitative metrics from the literature include an open-circuit voltage of 52.8 V, short-circuit current of 5.2 μA and maximum power density of 0.37 W/m^2^ for an optimized silk aerogel STENG, as reported in the study by Mi et al. [3]. Additionally, the STENG exhibited a lower power density of 4.3 mW/m^2^ [3].

Transitioning from dense 2D films to architectured 3D matrices basically alters the contact mechanics and stress distribution profiles of SF-TENGs. Even though conventional dense SF films suffer from limited interfacial contact and rigid deformation behavior, processing-induced hierarchical architectures, such as electrospun nanofibrous mats and freeze-cast aerogels, usually amplify the effective triboelectric contact area [7,17,155]. From a contact mechanics perspective, the ultralight, 3D interconnected porous network of SF aerogels allows for superior elastic compressibility and volumetric deformation under applied stress compared to planar films [7]. During compressive loading, the internal gaps within the aerogel matrices collapse, increasing the relative capacitance and enabling friction-induced electrification not only at the macroscopic interface but also across the vast interior pore surfaces [3,7]. Because of this, triboelectrically induced charges diffuse and reside stably within these internal cavities rather than dissipating, yielding remarkable improvements in electrical output, such as achieving power densities of 13.25 W/m^2^ in MXene-doped SF aerogels and 7.52 W/m^2^ in pure SF aerogels, considerably outperforming their dense film counterparts [2,7].

Adding silk fibroins as an additive material can improve the triboelectric output performance of other materials like CNF aerogels [3,84,85,86]. SF can function as an active functional material for energy harvesting [159], often serving as the tribopositive layer in device configurations [3]. Mi et al. highlights that its inherent tribopositivity also allows it to be used as an additive to enhance the performance of composite aerogels [3].

Advanced manufacturing methods provide additional control over structure–property–tribological interactions by introducing hierarchical surface roughness and nano-electrets. For instance, ESE technologies exploit atomized droplets to dissolve localized SF domains, creating quasi-periodically distributed nanopores guided by micro-patterned electric fields [155]. This targeted topological modification amplifies interfacial roughness and integrates hierarchical micro/nanostructures that boost triboelectric output by up to 2.6 times compared to smooth planar films [155]. Also, to withstand the thorough wear of continuous cyclic contact-separation, the mechanical durability of SF networks can be enhanced through the generation of in situ interlocked superelastic microfibers via co-solvent electrospinning-electrospraying [171]. Such processing delivers highly permeable and resilient matrices that distribute mechanical stress uniformly across the fibrous network, preventing serious material fatigue and maintaining stable electrical output over tens of thousands of deformation cycles [156,157,180,181]. Therefore, the strategic engineering of SF architectures (balancing multiscale porosity, elastic recovery, and β-sheet-driven wear resistance) serves as the conclusive mechanism for maximizing both tribological resilience and charge transfer efficiency in next-generation SF-TENGs.

## 6. Comparative Analysis of Natural Biopolymers for Triboelectric Applications

### 6.1. Comparative Assessment of Natural Biopolymers AM Integration

Despite the broad compatibility of cellulose, chitosan and silk fibroin with AM technologies, the literature reveals that each biopolymer presents distinct processing–structure–performance constraints that directly influence triboelectric functionality. In cellulose-based systems, the main limitation is associated with the need for strict rheological control during extrusion, since highly entangled nanofibrillar networks often require crosslinking strategies, viscosity modifiers or nanofiller incorporation to maintain print fidelity and structural stability [1,33,39,44,49,69,182,183,184,185].

CS-based systems exhibit favorable shear-thinning behavior and strong potential for biomedical interface engineering. Still, their printability and mechanical stability remain very dependent on hydrogel formulation, crosslinking density and moisture sensitivity [9,22,24,56,186,187,188,189,190].

Compared with cellulose and CS, SF presents a more complex conformational challenge because the printable Silk I state must subsequently transition into the mechanically stable Silk II β-sheet structure after deposition [10,69,87,162,190,191,192,193]. Even so, SF demonstrates particular versatility for multifunctional bioinks due to its tunable polymorphism, compatibility with multiple printing technologies and ability to stabilize sensitive biomolecules and bioactive additives [138,153,162,165,190,191,192,193,194].

Taken together, these observations indicate that the integration of natural biopolymers into AM-enabled triboelectric systems is governed not only by their biocompatibility or triboelectric behavior, but also by the balance between rheological processability, structural programmability, dielectric optimization and post-printing stability. To understand the behavior of each material in the relevant conditions of AM, the main processing techniques, rheological signatures and printing constraints identified in the literature have been summarized in Table 10.

Overall, the comparative analysis reveals that each biopolymer imposes distinct AM processing constraints arising from its intrinsic molecular organization and rheological behavior. Cellulose-based systems provide the highest printability stability due to their strong shear-thinning behavior and fibrillar reinforcement capability, whereas silk fibroin offers superior structural tunability but requires strict control over β-sheet crystallization during printing [10,22,56,62,195,196,197]. CS-based systems demonstrate excellent biocompatibility and ionic tunability, although their high viscosity and moisture sensitivity complicate large-scale printing fidelity and long-term dimensional stability.

### 6.2. Comparative Assessment of Surface Triboelectric Behavior Across Natural Biopolymer Systems

The surface triboelectric behavior of natural biopolymers is particularly linked to their chemical structure, surface energy and micro/nanoscale morphology. Cellulose, chitosan and silk fibroin all serve as prominent tribopositive materials in TENGs due to the abundance of electron-donating groups, such as hydroxyl (–OH), amine (–NH_2_), and amide linkages [1,3,99]. The triboelectric charge generation mechanism in these dielectrics is primarily governed by contact electrification and electrostatic induction, which can be described by the electron cloud potential model at the atomic level [1,198]. Upon physical contact with a more electronegative counter-layer (e.g., PTFE or PVDF), the overlap of electron clouds forms an asymmetric double potential well, driving electron transfer from the biopolymer to the counter-layer [198].

Nanoscale and microscale morphology, alongside porosity and surface roughness, exert profound effects on the triboelectric performance of these biopolymers. Increasing surface roughness generally amplifies the effective contact area and friction, leading to enhanced surface charge generation [50,199]. However, the role of porosity introduces a particular trade-off. In cellulose and silk fibroin aerogels, high porosity provides a vast internal surface area and structural compressibility that facilitates charge generation [3,7]. For instance, shifting from a 2D silk fibroin film to a 3D highly porous aerogel yields a 6.5-fold increase in voltage [7]. Conversely, excess free volume (air) within porous architectures can lower the effective dielectric constant of the material, as air has a relative permittivity of approximately 1, which may negatively impact the charge storage capacity of the matrix [1].

To overcome innate limits in charge density, dielectric behavior and polarity tuning are frequently employed through the incorporation of fillers, nanocomposites and chemical modifications. In cellulose systems, chemical grafting of functional groups with high dipole moments (e.g., –NO_2_) or fluorination (which flips the material to tribonegative) directly alters the molecular polarity and enhances the dielectric constant [1,69]. Chitosan systems benefit considerably from ionic coordination; the introduction of Mg^2+^ coordinates with chitosan chains to enhance intermolecular hydrogen bonding and dipole arrangements, increasing the surface potential from 895 mV to 965 mV and boosting power output without compromising biodegradability, as reported by He et al. [99]. Silk fibroin matrices demonstrate exceptional synergy with conductive or highly dielectric 2D fillers, such as MXene or MoS_2_ [172,175]. The addition of carbon nanotubes (MWCNTs) or quartz fibers to chitosan and cellulose not only reinforces mechanical integrity but also leverages interfacial polarization (Maxwell–Wagner relaxation) to significantly elevate the dielectric constant and short-circuit current [106,115].

When translated to AM, these surface-engineering strategies impose distinct constraints. Cellulose and chitosan inks used in extrusion-based 3D printing or DIW must exhibit pronounced shear-thinning behavior and high zero-shear viscosity to maintain shape fidelity [22,64]. During DIW, the shear and extensional flow can align anisotropic fillers like cellulose nanocrystals (CNCs), dictating the resultant microstructural properties [64]. Chitosan printing further relies on controlled solvent evaporation or post-printing neutralization baths to solidify the hydrogel, which can induce micro-wrinkled surface topographies beneficial for triboelectric contact [22]. Silk fibroin, instead, presents unique AM challenges; its propensity to form crystalline β-sheet structures can readily clog fine printing nozzles [190]. Because of this, silk is often modified into photo-crosslinkable derivatives (e.g., Sil-MA) for use in high-resolution vat DLP techniques, trading native structural simplicity for precise, nozzle-free patterning [197]. To facilitate a direct cross-material comparison, Table 11 summarizes the representative surface-engineering strategies, structural characteristics, and quantitative triboelectric performance metrics reported for cellulose, chitosan and silk fibroin-based TENG systems, together with their main functional advantages and limitations.

A comparative benchmarking of natural biopolymer TENGs reveals a clear performance hierarchy, with silk fibroin composites consistently achieving the highest power densities (up to 35.76 W/m^2^), followed by engineered chitosan and cellulose systems [51,173]. Silk’s superior performance derives from its notably high intrinsic tribopositivity and its structural synergy with conductive 2D nanofillers (e.g., MXene) in highly porous aerogel architectures, which provide vast internal surface areas for effective charge trapping [2,7]. Across all classes, dielectric engineering and nanostructuring act as the main performance drivers. The integration of high-*k* nanoparticles (e.g., BaTiO_3_ in cellulose) or ionic coordinators (e.g., bentonite in chitosan) markedly enhances interfacial polarization, boosting voltage and charge density [42,51]. Despite these quantitative gains, some limitations impede large-scale and long-term implementation. Highly porous 3D structures, while outstanding for maximizing contact area, suffer from mechanical degradation and structural collapse under continuous cyclic compression [7]. Also, the native hydrophilicity of unmodified natural biopolymers makes them particular susceptible to ambient humidity, which rapidly dissipates generated surface charges and limits their reliability in extreme or fluctuating environmental conditions [50,200].

For AM-enabled TENG interface engineering, future designs must carefully balance the enhancement of surface roughness and contact area with the potential drawbacks of increased free volume, ensuring that rheological printability constraints do not compromise the dielectric permittivity required for high-efficiency biomechanical energy harvesting. Table 12 summarizes the main advantages and limitations of the analyzed biopolymers used in TENGs.

Although biodegradable TENG systems have advanced rapidly, direct comparison across studies remains difficult due to the lack of standardized testing conditions, including variations in contact force, frequency, humidity, device geometry and electrode configuration. Also, many reports emphasize peak electrical performance without systematically evaluating long-term cyclic durability, biodegradation kinetics or the scalability of AM strategies.

## 7. Discussion

The development of TENGs from natural biopolymers represents a paradigm shift toward sustainable and self-powered bioelectronics. A critical synthesis of the current literature reveals that while cellulose, CS and SF all exhibit favorable tribopositive characteristics due to abundant electron-donating functional groups (like –OH, –NH_2_ and amide linkages), their optimal pathways for triboelectric enhancement fundamentally diverge. Cellulose heavily relies on chemical functionalization (e.g., fluorination, sulfonation) or dielectric filler incorporation to overcome its intrinsically weak surface polarity. CS, while possessing intrinsic antimicrobial properties and biodegradability, requires targeted modulation, such as metal-ion coordination (Mg^2+^) or the embedding of quartz fibers, to stabilize its intermolecular hydrogen bonding and elevate its relatively low native surface charge density. On the contrary, silk fibroin achieves the highest absolute performance ceilings among the reviewed materials primarily through profound structural re-engineering. By transitioning from two-dimensional films to three-dimensional highly porous nanocomposite aerogels, silk fibroin matrices (e.g., SF-MXene composites) can deliver remarkable power densities up to 9.92 W/m^2^ [172]. These distinct mechanistic dependencies underscore that there is no universal approach to optimizing biopolymer TENGs; rather, surface engineering must be strictly tailored to the native molecular architecture of a specific biomaterial.

AM serves as a transformative tool in this domain by granting precise spatial control over surface topography and hierarchical porosity, which directly govern contact electrification. However, integrating AM introduces severe, material-specific processing constraints that complicate triboelectric design. Extrusion-based printing of cellulose and chitosan imposes strict rheological parameters, necessitating pronounced shear-thinning behavior and rapid stabilization mechanisms, such as complex solvent exchange or dual-crosslinking schemes. Silk fibroin, on the other hand, presents a particularly acute structural challenge: the transition from the amorphous Silk I conformation (necessary for low-viscosity extrusion) to the crystalline β-sheet Silk II conformation (required for mechanical stability) frequently induces severe nozzle clogging. Because of this, fabrication is often restricted to photo-crosslinkable derivatives like Sil-MA in vat polymerization processes. Likewise, the potential of AM remains significantly underexploited. Although the current literature has successfully demonstrated the fabrication of bulk scaffolds and micro-wrinkled topographies, there is a glaring absence of complex multi-material triboelectric gradients or anatomically patient-specific geometries, thus emphasizing the need for future innovation.

A pervasive limitation across the reviewed literature is the inevitable compromise between maximizing triboelectric output and preserving the biomedical and mechanical integrity of the biopolymer. Strategies that exponentially increase specific surface area, such as the synthesis of ultralight, highly porous aerogels, fundamentally introduce a vast internal free volume of air. Because air possesses a relative permittivity of approximately 1, excessive porosity paradoxically diminishes the effective dielectric constant of the structural matrix, limiting overall charge storage capacity. Similarly, the aggressive chemical functionalization of cellulose to flip its polarity (e.g., via fluorination) or the heavy doping of chitosan with synthetic nanoparticles risks compromising the materials’ native non-toxicity, biodegradability and cytocompatibility. Moreover, the mechanical resilience of these biopolymers is frequently sacrificed for electrical gain; pure chitosan films suffer from high brittleness and low strength, requiring plasticization that subsequently alters the triboelectric interface. Achieving a rational balance between good mechanical flexibility, stringent biocompatibility and high dielectric performance remains an unresolved challenge.

Methodologically, the literature suffers from pronounced gaps, particularly regarding incomplete and inconsistent reporting. The current data are highly disjointed; reported power densities fluctuate drastically without standardized context, ranging from 42 mW/m^2^ [39] for bacterial cellulose/ZnO nanocomposites to 300 W/m^−2^ [1] for modulated cellulose and 7.52 W/m^2^ [7] for highly porous silk aerogels. As explicitly stated in the reviewed findings, the high heterogeneity in experimental setups, material formulations and evaluation metrics completely precludes reliable statistical meta-analysis. Surface topography parameters, such as roughness and porosity gradients, are frequently incompletely characterized alongside electrical outputs. Importantly, most of the current research is confined to benchtop proof-of-concept demonstrations, with a conspicuous lack of rigorous in vitro and in vivo validation assessing long-term degradation kinetics, dynamic mechanical stability and bio-integration in realistic physiological environments.

Advancement in AM triboelectric biointerfaces demands a standardization of testing protocols and reporting metrics (e.g., normalized *V_oc_*, *I_sc_* and power density relative to contact force and humidity) to enable objective cross-study comparisons. Future research could prioritize coupling advanced in situ surface metrology with TENG performance evaluation during the AM process, allowing for real-time monitoring of how layer-by-layer deposition alters dielectric properties. Engineering approaches must be directed toward more rational selection of a biocompatible filler and highly targeted functional group engineering that simultaneously preserve eco-friendly profiles and boost triboelectric charge density. In the end, bridging the gap from conceptual devices to clinically relevant wearable or implantable prototypes will necessitate comprehensive in vivo validation methodologies.

## 8. Current Challenges and Future Perspectives

While the integration of natural biopolymers—in this case cellulose, chitosan and silk fibroin—into AM TENGs has shown potential for sustainable energy harvesting and biointerface engineering, some barriers still impede their widespread commercialization and large-scale deployment.

### 8.1. Additive Manufacturing Challenges

The translation of natural biopolymers from traditional solvent-casting methods to advanced AM techniques remains hindered by underlying processing constraints. Foremost among these is rheological instability. Extrusion-based 3D printing and DIW require bioinks that display specific non-Newtonian behaviors, such as: pronounced shear-thinning to ensure smooth flow through micro-nozzles, followed by a rapid recovery of yield stress to maintain high print fidelity and prevent structural collapse post-deposition [43,56,59]. Formulating pure cellulose inks often demands complex and harsh solvent systems (such as NaOH/urea or ionic liquids) or the utilization of highly concentrated nanofibril suspensions, which can lead to unpredictable viscoelastic properties and dimensional contraction during solvent evaporation or coagulation [12,59].

CS hydrogel inks encounter similar rheological bottlenecks. The necessity for post-printing neutralization baths to induce gelation frequently results in poor print fidelity, significant volumetric contraction and structural warping [43,119]. SF presents perhaps the most particular and challenging AM processing profile. The high shear stress experienced within narrow printing nozzles can prematurely trigger the conformational transition of silk from random coils to highly crystalline sheet structures, resulting in rapid, uncontrolled gelation and marked nozzle clogging [192]. Also, attempting to enhance the triboelectric output of these biopolymers through the integration of functional additives (e.g., CNTs, MXene, or high-*k* nanoparticles) introduces important filler dispersion challenges. Highly viscous biopolymer inks somehow restrict the uniform dispersion of these nanomaterials, leading to localized agglomeration. This uneven distribution not only compromises the rheological consistency required for reproducible printing but also creates macroscopic defects that undermine the structural integrity and scalability of the printed TENGs [42,59,201].

### 8.2. Surface Engineering and Dielectric Modulation Limitations

To maximize the TECD of biopolymers, researchers often employ surface micro-structuring and dielectric modulation. Even so, these strategies introduce several mechanical and electrical compromises. While dispersing high-*k* dielectric fillers (such as BaTiO_3_) or conductive nanomaterials enhances interfacial polarization and charge storage, excessive filler loadings inevitably lead to filler agglomeration [42,63]. This agglomeration disrupts the continuous polymeric network, introducing interfacial instability and structural defects that worsen dielectric loss and diminish the overall energy conversion efficiency [123].

Moreover, engineering highly porous 3D architectures, such as aerogels and nanofibrous sponges, properly maximizes the specific surface area for contact electrification [7]. This increased free volume noticeably compromises the mechanical stability of the TENG. Under prolonged, cyclic mechanical compression, these hierarchically porous networks are very susceptible to structural collapse, plastic deformation and major cyclic degradation, ultimately limiting their long-term charge retention and operational lifespan [7,48].

It should also be noted that chemical surface modifications aimed at tuning triboelectric polarity often induce significant brittleness into the naturally rigid biopolymer chains [97,104]. Still, the most particular limitation across all natural biopolymers is humidity-induced charge dissipation. The abundant hydrophilic functional groups (e.g., –OH and –NH_2_) characteristic of cellulose, CS and SF rapidly adsorb ambient water molecules via hydrogen bonding. This adsorbed moisture forms a conductive surface layer that quickly loses the generated electrostatic charges and screens the contact electrification process, rendering the TENG significantly unreliable in varying environmental conditions [47,104,109].

### 8.3. Comparative Material-Specific Limitations

A comparison of the three main biopolymer systems shows that particular material-specific limitations can be observed that affect their processing and application boundaries:

- Despite being abundant, the thermal stability and mechanical stiffness of native cellulose occupy a nearly neutral position in the triboelectric series, resulting in a characteristically low charge density [12,48,54]. To render cellulose highly tribopositive or tribonegative, complex chemical functionalization (such as amination, sulfonation, fluorination or cyanoethylation) is required [42]. In addition, its moisture sensitivity may degrade its electrical output in ambient environments, necessitating advanced hydrophobic surface treatments that complicate AM processing [12,54].
- Since CS contains many free primary amine groups, it exhibits a more naturally tribopositive character than cellulose [101]. Still, CS films and hydrogels are characteristically plagued by high mechanical brittleness and an exceptionally high water affinity [97,104]. Although incorporating plasticizers (e.g., glycerol) can enhance its flexibility and wearability for electronic skin applications, excessive plasticization disrupts the intermolecular hydrogen-bonding networks, which can inadvertently lower the material’s dielectric constant and significantly degrade its triboelectric output performance [89].
- SF usually achieves the highest baseline triboelectric output among the three biopolymers owing to its outstanding electron-donating capabilities and dense amide linkages [153,173]. The most important limitation of silk is its notable processing complexity [2]. The structural performance of silk is entirely dependent on the ratio of random coils to sheet crystals. While a higher sheet content improves water resistance and mechanical resilience, it concurrently markedly reduces the material’s flexibility, complicates extrusion-based 3D printing and decreases the surface conformability required for efficient triboelectric contact electrification [153,160,192].

### 8.4. Standardization Issues

One impediment to the systematic advancement of biopolymer-based TENGs is the pervasive lack of standardized testing protocols across the literature [42]. Today, studies inconsistently report core output metrics, such as open-circuit voltage (*V_oc_*), short-circuit current (*I_sc_*), and power density, often without normalizing these values to the specific contact area or internal impedance of the device. Also, there is a critical lack of standardized testing conditions. The triboelectric output is hypersensitive to extrinsic environmental and physical variables, including relative humidity, applied mechanical force, contact frequency, device geometry and the electron affinity of the chosen reference counter-electrode (e.g., PTFE vs. PDMS) [42,97]. Without establishing generally accepted performance metrics and standardized calibration environments, accurately decoupling existing material properties from imposed testing conditions remains nearly impossible, preventing precise cross-material benchmarking and hindering the establishment of reliable structure–property–performance relationships [42].

### 8.5. Future Perspectives

To overcome these challenges and facilitate the full potential of next-generation biodegradable TENG technologies, some strategic research directions must be pursued:

- Research must transition from laboratory-scale manual casting to high-throughput, scalable AM techniques [2,41,48]. Developing photocurable biopolymer derivatives (e.g., methacrylated silk or carboxymethyl cellulose) for use in high-resolution DLP or stereolithography can avoid the rheological limitations of extrusion, allowing accurate, nozzle-free fabrication of complex, hierarchical micro-architectures [121].
- Future material designs must refine dielectric engineering by seamlessly integrating advanced 2D nanomaterials (such as MXene, MoS_2_, or functionalized graphene) and core–shell nanostructures into the biopolymer matrix [12,54,172]. These multifunctional nanocomposites may optimize interfacial polarization and trap states to maximize charge density, while reducing filler agglomeration, mechanical degradation and dielectric loss [12,54].
- A particular opportunity can be found in the development of environmentally stable, fully biodegradable TENGs for continuous human health monitoring [12,109,153]. Advanced surface hydrophobization strategies (e.g., silanization or the integration of natural hydrophobic waxes) must be optimized to repel moisture without compromising the material’s biocompatibility. This will pave the way for self-powered, bioresorbable implantable sensors that can operate safely in vivo and degrade harmlessly after their operational lifespan, thereby eliminating secondary electronic waste [153].
- The integration of 4D printing introduces the smart concept, self-morphing triboelectric structures [58,202]. By designing biopolymers in order to adapt their macroscopic geometry in response to specific environmental stimuli (such as temperature, pH or moisture), TENGs can significantly self-optimize their contact mechanics and surface area, leading to very adaptive, self-regulating energy harvesting systems.
- Given the broad parameter space involving ink rheology, functional group density, dielectric constant and structural porosity, the integration of artificial intelligence (AI) and machine learning (ML) algorithms is important [42,48,50,60,109,110]. Data-driven, AI-assisted material optimization can swiftly screen complex biopolymer formulations and predict triboelectric outputs, linking the gap between molecular surface engineering and macroscopic device performance.

Overall, although cellulose, CS and SF each present distinct rheological and triboelectric limitations, their highly customizable surface chemistries and good biocompatibility make them promising biomaterials for the future of green electronics. Through the development of scalable AM technologies, standardization of TENG evaluation metrics and use of AI-driven dielectric engineering to overcome moisture and durability constraints, the realization of stable, fully biodegradable, self-powered biointerfaces can be actualized.

## 9. Conclusions

The integration of natural biopolymers with AM and advanced surface-engineering strategies represents a conceptual shift in the development of sustainable, self-powered TENGs for biomedical applications. This comprehensive review highlights that while cellulose, chitosan and silk fibroin all possess good biocompatibility and baseline tribopositivity, their optimal pathways for triboelectric enhancement are in essence distinct. Cellulose relies predominantly on chemical functionalization (e.g., sulfonation, fluorination) and the incorporation of dielectric fillers to overcome their intrinsically weak surface polarity and increase deep trap density. Chitosan’s relatively low native charge density is effectively elevated through metal-ion coordination (e.g., Mg^2+^) and the embedding of conductive or structural micro-fillers, which simultaneously reinforce its innate brittleness and promote interfacial polarization. SF shows the highest potential for absolute performance, reaching impressive power densities after transitioning from 2D films to 3D nanocomposite aerogel structures. The overarching mechanistic theme for each of these three biopolymer materials appears to be the way in which surface roughness directly impacts contact electrification and electrostatic induction. Furthermore, dielectric control, chemical functionalization and filler materials have been shown to be of critical importance for altering the polarity of the materials to achieve maximal charge generation and capacity. AM has shown promise in this field by allowing for microarchitectural control of the vital micro-architectures that regulate these critical surface topographies. After reviewing the current state of the field, we have identified several methodological limitations. The heterogeneity of the current state of the field makes it difficult to compare studies, with outputs fluctuating drastically depending on the testing scenario.

To overcome these challenges, future research directions must be focused on critical areas based on the present knowledge base. Firstly, there is a significant need for standardization of the triboelectric measurement protocols and results, where the results in the form of open-circuit voltage, short-circuit current and power density need to be normalized based on factors such as contact pressure and humidity. High-resolution in situ surface analysis during AM processes will be mandatory for continuous monitoring of the effects of layer-by-layer deposition and microstructural changes on the overall performance of the devices. AM techniques must be advanced from uniform tissue engineering scaffolds to the creation of complex multi-materialized biointerfaces and gradients that can mimic the exact anatomy and gradients of the body. Since the vast majority of the present research is at the benchtop proof-of-concept level, in vitro and in vivo validation must be prioritized for the assessment of long-term degradation kinetics, dynamic mechanical stability and physiological integration. Bioink formulations must be focused on rational filler selection and functional groups for successful triboelectric enhancement while meeting stringent sustainability and biodegradability criteria.

To conclude, it is worth emphasizing that the convergence of surface tribology, biomolecular engineering and AM positions cellulose, chitosan and silk fibroin as indispensable materials for the future of eco-friendly bioelectronics. By resolving existing methodological inconsistencies and navigating the complex trade-offs between dielectric performance, printability and biological safety, the rational design of these biopolymer systems will catalyze the transition of sustainable TENGs from fundamental benchtop research into strong, clinically relevant prototypes for advanced biomedical interface engineering.

## Figures and Tables

**Table 1:** Processing-induced structural features in cellulose-based triboelectric systems.

Cellulose Form	Primary Processing Route	Structural Morphology Obtained	Physicochemical Characteristic	Functional Implication for TENG Behavior	Ref.
Cellulose II/Cellulose Nanofiber (CNF)	Dissolution–regeneration (e.g., NaOH/urea, ionic liquids) and freeze-drying	3D interconnected hierarchical porous network (aerogel)	High specific surface area and macroscopic structural elasticity	Mechanically amplifies relative capacitance and maximizes effective interfacial contact area under cyclic compressive stimuli	[1,34,41,42]
CNF/Cellulose Nanocrystal (CNC)/Cellulose Inks	Extrusion-based additive manufacturing (direct ink writing)	3D micro/nano hierarchically patterned architectures with programmed geometries	Shear-induced nanofibrillar alignment and viscoelastic shape fidelity	Markedly amplifies effective triboelectric contact area and ensures mechanical resilience against structural fatigue	[42,43,44]
Cellulose Acetate (CA)/Cellulose Ethers	Electrohydrodynamic processing (electrospinning)/solution blowing	Ultra-porous, nonwoven nanofibrous webs	Exceptional surface-area-to-volume ratio and tunable multiscale roughness	Enhances charge-trapping capability and contact electrification efficiency while maintaining structural flexibility and breathability	[1,44,45]
Cellulose Fibers/Cellulose Paper	Mechanical creping/template-assisted molding (e.g., sandpaper imprinting, leaf venation)	Wrinkled macroscopic architectures with programmed spatial wavelengths and amplitudes	High topographical compliance and enhanced microroughness	Optimizes wave-driven contact-separation dynamics, conforms to soft counter-layers, and amplifies friction-induced charge generation	[1,41,44,45]
Cellulose Composites with 0D-2D Fillers	Physical doping/liquid-phase exfoliation/co-precipitation	Embedded 3D network of metallic, high-*k*, or carbonaceous nanofillers	Formation of dense microcapacitors and intensive interfacial polarization	Lowers intrinsic impedance, triggers coupled power output surges and accelerates electron transfer while restricting leakage currents below the percolation threshold	[1,42,46]
Covalently Functionalized Nanocellulose	Surface chemical engineering (e.g., sulfonation, fluorination, amination, silanization)	Molecular-scale functionalized backbone without disrupting core fibrillar morphology	Alteration of local electronic band structure, engineered deep traps and shifted surface potential phase	Significantly shifts tribo-polarity, expands hole deep trap density and systematically suppresses the kinetic dissipation of trapped triboelectric charges into the environment	[1,4,12]

**Table 2:** Additive manufacturing strategies for cellulose-based TENG architectures.

AM Technique	Printable Cellulose Formulation	Rheological Requirement	Structural Advantage	Main Processing Limitation	Triboelectric Relevance	Ref.
Direct Ink Writing (DIW)/Microextrusion	Cellulose nanofiber (CNF) hydrogels and partially dissolved cellulose suspensions	Shear-thinning behavior with rapid viscoelastic recovery to maintain extrusion stability and prevent pattern collapse	Fabrication of hierarchical porous architectures with programmable geometries and fibrillar alignment	Post-print treatments (e.g., freeze-drying, solvent exchange) may induce contraction and structural collapse, reducing shape fidelity	Enhances compressibility and effective interfacial contact area during cyclic deformation, resulting in higher output compared to planar structures	[41,43,56,58,59]
Extrusion-Based 3D Printing (Conductive/Dielectric Inks)	Ethyl cellulose (EC) or carboxymethyl cellulose (CMC) blended with conductive or dielectric fillers (e.g., CNTs, hBN)	Yield-stress behavior and controlled rheology are required to ensure filament stability and homogeneous filler dispersion	Enables spatial integration of conductive pathways and localized dielectric phases within 3D architectures	High filler loading may cause nozzle clogging and reduced ink extrudability	Modulates dielectric polarization and facilitates charge transfer through embedded conductive and dielectric networks	[44,49]
Electrohydrodynamic Processing (Electrospinning)	Cellulose acetate (CA), cyanoethyl cellulose (CEC), or nanocellulose combined with dielectric polymers	Sufficient polymer chain entanglement and viscosity are required to maintain stable jet formation during spinning	Produces porous nanofibrous membranes with high surface area and tunable roughness	Limited capability for generating controlled 3D macroscopic geometries and dependence on solvent evaporation dynamics	Enhances contact electrification efficiency through increased surface area, porosity and charge-trapping capability	[44,45,60,61]

**Table 3:** Surface engineering and dielectric modulation strategies in cellulose-based TENGs.

Surface Modification Strategy	Mechanism of Action	Structural/Surface Effect	Electrical Effect	Main Drawback	Ref.
Deep Trap Engineering (Sulfonation)	Substitution of hydroxyl groups with sulfonic acid (–SO_3_H) functionalities on the cellulose backbone	Modifies local surface electronic structure while preserving fibrillar morphology at controlled substitution levels	Increases deep trap density and dielectric constant, improving triboelectric charge retention	Excessive substitution reduces cellulose crystallinity and mechanical integrity	[4]
Tribo-Polarity Reversal (Fluorination/Silanization)	Surface grafting of fluorinated silanes with strong electron-withdrawing capability	Increases nano-roughness and surface hydrophobicity	Shifts surface potential toward negative polarity and enhances electron-capturing capability	Multi-step processing and reduced environmental sustainability due to fluorinated compounds	[42,69]
Electron-Donation Enhancement (Amination)	Grafting of amino (–NH_2_) groups through etherification, crosslinking or aminosilane coupling	Reconstructs hydrogen-bonding networks and modifies surface wettability	Enhances electron-donating capability and promotes positive surface polarization	Requires complex synthesis routes and potentially toxic crosslinking agents	[1,42]
Dielectric Modulation (High-*k* Nanoparticles)	Incorporation of high-*k* nanoparticles (e.g., BaTiO_3_, Fe_3_O_4_) to form localized microcapacitor networks	Increases structural heterogeneity and localized defect density	Enhances interfacial polarization and equivalent capacitance	High filler loading may induce agglomeration, charge leakage and electrical breakdown	[1,46]
Conductive Network Integration (AgNWs, PPy, CNTs)	In situ polymerization or physical incorporation of conductive phases within the cellulose matrix	Enhance internal conductivity and localized surface roughness	Facilitates charge transfer and electrostatic induction	Excessive conductive loading may cause short-circuiting and reduced biocompatibility	[1,34,44,70]
Hierarchical Micro/Nano-Texturing	Surface structuring through lithography, creping, template-assisted molding or 3D printing	Produces porous or wrinkled architectures with increased deformability	Enhances frictional contact area and triboelectric charge generation	Surface microstructures may undergo abrasive wear and cyclic fatigue	[34,45,63]

**Table 4:** Processing-induced structural features in chitosan-based triboelectric systems.

Chitosan Form	Primary Processing Route	Structural Morphology Obtained	Physicochemical Characteristics	Functional Implication for TENG Behavior	Ref.
Semicrystalline Dense Film	Solution casting with pH/solvent modulation	Compact morphology with dense polymer chain packing	Controlled molecular chain arrangement (c-axis) and varied degree of surface protonation (NH_3_^+^)	Crystallization shifts triboelectric polarity, while excessive acidification may induce charge screening and structural collapse	[50,104]
Polymeric Nanocomposite	Nanoparticle incorporation	Matrix-embedded dispersion with localized phase boundaries	Increased dielectric permittivity and interfacial heterogeneity	High-*k* nanofillers promote interfacial charge trapping and restrict electrostatic dissipation; excessive loading may induce agglomeration and leakage currents	[51,104,105,106]
Nanofibrous Structure	Electrospinning	Interconnected nonwoven fibrillar network	High surface area and mechanical compressibility	Enhanced structural deformation increases effective contact area and electrostatic interactions; limited cyclic durability without secondary plasticization	[106,107]
Micro-Patterned Membrane	Soft lithography/templating	Hierarchical anisotropic microstructures	Increased surface roughness and topographical heterogeneity	Surface protrusions enhance contact deformation and triboelectric interactions; excessively coarse templates reduce active contact density	[108,109]
Chemically Modified Derivative	Chemical crosslinking/surface functionalization	Continuous film with covalently modified side groups	Altered hydrogen bonding and dipole distribution	Enhanced dipole polarization and triboelectric polarity with improved moisture resistance; requires complex synthesis routes	[97,110]
Dual-Network Hydrogel	Dynamic covalent crosslinking	Hydrated interconnected porous matrix	High stretchability, ionic conductivity and stress relaxation	Ionic mobility enhances interfacial polarization and capacitance; high moisture sensitivity reduces long-term stability	[111,112]
Ion-Doped Composite	Ion embedding (e.g., CaCl_2_ addition)	Ion-coordinated polymer matrix	Increased ionic mobility and localized structural flexibility	Ion coordination enhances dipole density and contact electrification; excessive salt loading induces hygroscopicity and rheological instability	[113]

**Table 5:** Additive manufacturing strategies for chitosan-based TENG architectures.

AM Technique	Printable Chitosan Formulation	Rheological Requirement	Structural Advantage	Main Processing Limitation	Triboelectric Relevance	Ref.
Extrusion-Based DIW	Pure chitosan dissolved in multi-acidic mixtures followed by alkaline neutralization	Shear-thinning behavior with rapid viscosity recovery to maintain extrusion stability	Flexible micro-fibrous networks with wrinkled and porous morphologies	Drying-induced shrinkage, nozzle clogging at high viscosities, and structural collapse without controlled gelation	Hierarchical roughness increases effective contact area, while acid-base neutralization modulates surface charge density	[22,51,119]
Electrohydrodynamic Processing (Electrospinning)	High-molecular-weight chitosan blended with plasticizers or processed in concentrated acetic acid	Polymer concentration must ensure stable jet formation and prevent bead defects	Interconnected nonwoven nanofibrillar architectures with high compressibility and large solid–air interfaces	Restricted solubility window, limited cyclic durability and high humidity sensitivity	Enhanced deformation and contact area improve electrostatic induction and charge transfer	[103,108]
Vat Photopolymerization (DLP)	Methacrylated chitosan with photoinitiators or bio-derived photoactive dyes	Low prepolymer viscosity and rapid photo-curing response are required	High-resolution anisotropic 3D geometries with high hydration capability	Complex synthesis, limited crosslinking efficiency, and reduced long-term stability under excessive hydration	Enables controlled geometries for tailored contact mechanics, although moisture retention suppresses charge accumulation	[119,121]
Templated Biofabrication (Reverse Molding)	Chitosan matrix containing dual plasticizers (e.g., glycerol and polyethylene glycol)	Moderate viscosity and sufficient flowability for accurate template replication	Hierarchical anisotropic micro-asperities integrated into flexible films	Limited resolution due to surface tension and reduced mechanical strength under excessive plasticization	Surface protrusions and plasticizer-induced polarity shifts enhance triboelectric output	[89,108,109]
Hybrid Composite Extrusion Printing	Chitosan hydrogels doped with inorganic or organic particulate fillers	Optimized filler loading is required to preserve shear-thinning and enhance storage modulus	Improved compressive modulus, mechanical resilience, and shape fidelity	Excessive filler loading induces agglomeration, brittle fracture, nozzle occlusion, and leakage currents	Fillers enhance interfacial polarization, charge trapping, and TENG output density	[10,115]

**Table 6:** Surface engineering and dielectric modulation strategies in chitosan-based TENGs.

Surface Modification Strategy	Mechanism of Action	Structural/Surface Effect	Electrical Effect	Main Drawback	Ref.
Dielectric Nanofiller Incorporation (e.g., SWCNTs, MWCNTs, AgNWs, Clay)	Maxwell–Wagner relaxation at filler–biopolymer interfaces creates heterogeneous dielectric boundaries	Increased micro-roughness and interfacial heterogeneity within the polymer matrix	Enhances interfacial charge trapping, dielectric constant and contact electrification	Excessive filler loading induces agglomeration, leakage currents and mechanical embrittlement	[104,106,123]
Chemical Functionalization (e.g., Quaternization, Tannic Acid Grafting)	Covalent modification introduces electron-donating or electron-withdrawing functional groups	Alters hydrogen bonding, surface potential and electron affinity	Enhances dipole orientation and dielectric constant or reverses triboelectric polarity	Complex synthesis and sensitivity to pH and substitution degree	[97,110]
Ionic Modulation & Salt Embedding (e.g., Mg^2+^, Ca^2+^, NaCl Doping)	Metal cations coordinate with hydroxyl and amino groups of chitosan	Modifies hydrogen bonding, structural organization and surface roughness	Enhances dipole density, ionic mobility and surface charge density	High salt loading causes hygroscopicity, rheological instability and charge screening	[99,113]
Hierarchical Micro/Nanostructuring (e.g., Reverse Templating, Electrospinning)	Geometric surface structuring through templating or electrohydrodynamic processing	Produces porous fibrillar architectures and anisotropic microstructures	Increases contact area and electrostatic induction during deformation	High-porosity structures are susceptible to fatigue, collapse and moisture entrapment	[108]
Dual-Plasticizing & Polymeric Blending (e.g., Glycerol/PEG, Lignin)	Addition of plasticizers or secondary polymeric chains increases chain mobility and polar domains	Reduces Young’s modulus and improves conformal flexibility	Enhances surface electro-positivity and restricts triboelectric charge dissipation	Over-plasticization reduces tensile strength and dimensional stability	[89,95]

**Table 7:** Processing-induced structural features in silk fibroin-based triboelectric systems.

Silk Fibroin (SF) Form	Primary Processing Route	Structural Morphology Obtained	Physicochemical Characteristics	Functional Implication for TENG Behavior	Ref.
Dense 2D Planar SF Film	Spin coating/solvent casting followed by alcohol or water vapor annealing	Smooth, dense 2D planar layer	Transition from random coil to β-sheet crystal structure; water-insoluble and transparent	Amide groups provide electron-donating ability, although limited contact area restricts charge density; high humidity reduces cyclic stability	[152]
SF Nanofibrous Mats	Electrospinning	1D nonwoven, interconnected nanofiber network	High surface-to-volume ratio and C–O–N–H dipole alignment along fiber axis	Increased contact area enhances contact electrification and apparent piezoelectricity; high flexibility but limited fatigue durability	[153,154]
Highly Porous SF Aerogels	Directional freeze-drying/ice-templating	3D porous micro/nanofibrillated network	Ultralight structure with high compressibility and large internal surface area	Structural deformation enhances capacitance and charge trapping; excessive compression may induce structural collapse	[7]
Micro/Nano-architectured SF Films	Water electrospray-etching (ESE)	Hierarchical nanoporous surface	Localized dissolution of water-soluble domains and integration of nano-electrets (e.g., SiO_2_)	Increased surface roughness and improved charge retention; fabrication complexity limits large-scale processing	[155]
SF/Carbon Nanotube (CNT) Composites	Solution blending/electrospinning + electrospray	3D bridging network with CNTs wrapping or interconnecting SF fibers	Conductive percolation pathways with strong mechanical interlocking	Facilitates rapid charge transfer and improves flexibility; excessive CNT loading causes agglomeration and unstable output	[17,136]
MXene-Doped SF Composites	Vacuum filtration/solution mixing	Lamellar and biomimetic composite structure	Electronegative functional groups (–O, –F, –OH) and extensive hydrogen bonding with SF	Enhances surface polarity and power density; excessive MXene loading induces embrittlement and performance degradation	[17]
Ion-Doped SF Films (e.g., Li^+^, His)	Solution doping and casting	Flexible smooth film with disrupted hydrogen bonding	Enhanced dipole reorientation, ionic polarization and dielectric constant	Improves output voltage and current; salt hygroscopicity promotes charge dissipation under high humidity	[156]

**Table 8:** Additive manufacturing strategies for silk fibroin-based TENG architectures.

AM Technique	Printable Silk Fibroin Formulation	Rheological Requirement	Structural Advantage	Main Processing Limitation	Triboelectric Relevance	Ref.
Direct Ink Writing (DIW)/Extrusion	Concentrated aqueous SF or SF with rheological modifiers (e.g., Konjac gum, cellulose)	Shear-thinning behavior with rapid viscoelastic recovery for filament stability	Hierarchical 3D lattices with interconnected macropores and high deformability	High viscosity may induce nozzle clogging; solvent removal can compromise structural integrity	Macroporous architectures increase compressibility, contact area and electrical output	[162,163]
Digital Light Processing (DLP)	Methacrylated SF (Sil-MA) with photoinitiators and conductive dopants	Low-viscosity formulation enabling rapid photo-curing under UV/visible light	High-resolution anisotropic architectures with tunable mechanical stability	Photoinitiator toxicity, limited light penetration and post-curing brittleness	Conductive dual-networks enhance interfacial polarization and charge transfer	[168,169,170]
Synchronous Electrospinning & Electrospraying	SF dissolved in formic acid with nanoparticles or liquid metal inks	Controlled viscosity and surface tension to ensure stable fiber formation	Interlocked superelastic nanofibrous networks with high permeability	Solvent evaporation and mismatch between SF and conductors may induce phase instability	High surface area enhances contact electrification, while nano-electrets improve charge retention	[171]
Water Electrospray-Etching (ESE)	Water-soluble SF films with optional SiO_2_ nano-electrets	Controlled droplet atomization and dissolution kinetics	Hierarchical nanoporous surface topologies	Requires precise electric field calibration; porous structures remain humidity-sensitive	Surface roughness and nano-electrets improve contact area and charge retention	[155]
Soft Imprinting Lithography (SIL)	Regenerated aqueous SF cast onto patterned elastomeric molds	Low viscosity and sufficient fluidity for conformal mold wetting	Ordered 3D micro-architectures with increased specific surface area	Structural shrinkage and collapse during alcohol annealing limit scalability	Micro-patterned surfaces improve deformability and triboelectric charge generation	[157,160]

**Table 9:** Surface engineering and dielectric modulation strategies in silk fibroin-based TENGs.

Surface Modification Strategy	Mechanism of Action	Structural/Surface Effect	Electrical Effect	Main Drawback	Ref.
Ionic Modulation and Amino Acid Doping	Li^+^ disrupts hydrogen bonds to enhance ionic polarization, while Histidine promotes interfacial polarization between crystalline and amorphous regions	Modifies intermolecular spacing and induces internal dipole heterogeneity while maintaining a smooth surface	Increases dielectric constant and electron-donating/absorbing differential, greatly improving power density	Hygroscopic dopants induce moisture sensitivity and rapid charge dissipation at high humidity	[156]
2D MXene (Ti_3_C_2_T_x_) Nanosheet Incorporation	Electronegative surface groups (–O, –F, –OH) promote hydrogen bonding, interfacial polarization and conductive pathways	Produces porous, wrinkled, biomimetic lamellar architectures	Enhance surface charge density, contact electrification and reduce internal impedance	Excessive loading causes agglomeration, dielectric loss and charge leakage	[172,173]
Water Electrospray-Etching (ESE) + Nano-Electrets	Localized dissolution of SF domains under micro-patterned electric fields; SiO_2_ nanospheres act as charge-trapping nano-electrets	Generates hierarchical nanopores with increased surface roughness	Increases contact area, charge trapping and prolongs charge retention	Water-soluble etched domains remain unstable under humid conditions without methanol annealing	[155]
Electrospinning with Fluorinated Nanoparticle Electrospraying	Fluorinated SiO_2_ nanoparticles are anchored onto electrospun fibers and act as electron-withdrawing nano-electrets	Forms interlocked superelastic fibrillar networks with amplified roughness	Enhances contact area and deep-trap charge retention during deformation	Surface tension and modulus mismatch complicate scalability; humidity suppresses charge retention	[171]
3D Conductive Carbon Nanotube (CNT) Network Integration	CNT arrays establish conductive percolation pathways for rapid charge transport	SF-coated CNTs form porous vertically aligned 3D bridging networks	Enhances contact electrification and minimizes internal impedance	Excessive SF coating blocks pores and reduces effective contact area	[17]
Crystallinity Modulation via Alcohol/Vapor Annealing	Solvent or vapor treatment induces transition from random coils to β-sheet nanocrystals	Produces crystalline water-insoluble structures with improved stability	Stabilizes dipole alignment and improves environmental stability	Excessive β-sheet crystallization reduces dipole mobility and increases brittleness	[154,157]
Surface Dipole and Work Function Engineering (SMP Doping)	Electron-donating silk microparticles shift the work function and reduce electron transfer barriers	Introduces localized morphological and interfacial heterogeneity	Enhances surface potential and triboelectric charge transfer	Excessive filler loading reduces flexibility and mechanical integrity	[174]
2D Transition Metal Dichalcogenide (MoS_2_) Integration	Non-covalent interactions modulate dielectric permittivity and photo-stimulated charge dynamics	Enables homogeneous nanosheet dispersion and reduced structural defects	Enhances dielectric constant, lowers resistance and improves light-assisted output	Excessive loading causes nanosheet agglomeration and unstable electrical performance	[175]

**Table 10:** Comparative overview of AM processing parameters and printability of natural biopolymer systems.

Biopolymer System	Processing Technique	Ink Type and Solvent	Rheological Features	Crosslinking/ Stabilization	Printing Conditions/ Constraints	Geometrical Complexity/ Resolution	Ref.
Cellulose (cotton cellulose)	Extrusion 3D printing	6.3–6.7 wt% cellulose in DMSO/TBAH/H_2_O (8:1:1)	Solid-like at room temperature, strong shear-thinning	Solvent exchange (H_2_O) + chemical crosslinking (MBA)	Room temperature, 564 µm nozzle, 2 mm/s speed, 30 µL/s injecting speed	Complex 3D patterns, stable dimensions	Hu et al. [195]
Cellulose (cotton pulp/filter paper)	Direct ink writing (DIW)	3–10 wt% cellulose in NaOH/urea or NaOH/urea/ZnO	Thixotropic, shear-thinning, instantaneous self-support	Water bath coagulation (solvent removal), freeze-drying	Room temperature, 110–1070 µm nozzles, 1–10 mm/s speed, 10–800 kPa	Honeycombs, anatomical shapes/~250 µm resolution	Yuan et al. [56], Jiang et al. [58]
Cellulose (methacrylated CMC + CNCs)	Digital Light Processing (DLP)	Aqueous m-CMC and nanocrystals + BAPO-OH photoinitiator	n.r. *	Photo-crosslinking	n.r.	Lattice vascular networks, complex 3D parts	Cafiso et al. [62]
Cellulose (cellulose nanocrystals)	DIW/Inkjet	Aqueous CNC dispersion/gel	High viscosity, shear-thinning	Used as sacrificial support, water-removable	n.r.	Complex structures (boxes, spirals)	Li et al. [196]
Chitosan	Extrusion/DIW	6–10 wt% chitosan in acetic, lactic and citric acid mixture	Shear-thinning, Newtonian at low shear	Solvent evaporation, neutralization	Room temperature in air, 100–510 µm nozzles, 0.4–4.1 MPa pressure	3D scaffolds, starfish, leaf/~30 µm resolution	Wu et al. [22]
Chitosan/silk (chitosan + silk particles)	Extrusion	4 wt% chitosan in acidic solvent + milled silk particles	Shear-thinning, yield stress	Coagulation bath (NaOH/ethanol)	20 °C	Scaffolds with well-defined pores, reduced shrinkage	Zhang et al. [10]
Chitosan (phenolated chitosan + nanofibers)	Extrusion	Aqueous	High viscosity at low shear rates	Enzymatic (HRP-mediated)	n.r.	Tubular and nose structures with high fidelity	Sakai et al. [87]
Silk fibroin (Sil-MA)	DLP bioprinting	Aqueous Sil-MA + LAP photoinitiator	Low-viscosity liquid	Photo-crosslinking (UV 365 nm)	Room temperature, 50 µm thickness, 4 s curing per layer	Trachea, brain, ear/down to 200 µm resolution	Kim et al. [197]

* n.r. indicates “not reported” in the referenced source/s.

**Table 11:** Comparative benchmarking of triboelectric performance and surface-engineering strategies in cellulose, chitosan and silk fibroin-based TENG systems.

Biopolymer	Modification Strategy	Structural Morphology	*V_oc_* (V)	*I_sc_* (µA)	Output Metric	Key Advantage	Key Limitation	Ref.
Cellulose	Amine functionalization and polymer blending	Composite paper	222.1	4.3	39.7 µC/m^2^	High electron-losing ability and facile fabrication	Output performance degrades under high relative humidity	Lin et al. [200]
Cellulose	High-*k* nanoparticle doping	Nanofibrous composite paper	170	9.8	n.r. *	Enhanced dielectric constant improves surface charge density	Filler wear and friction reduce long-term durability	Fernandes et al. [35]
Chitosan	Natural clay decoration (1 wt% bentonite)	Composite biofilm	996	n.r.	26.5 W/m^2^	High power density achieved using biocompatible additives	Excess additive loading reduces performance stability	Yar et al. [51]
Chitosan	Carboxylic acid doping (citric acid)	Flexible solid film	157	53	45.5 W/cm^2^	Flexible network obtained through simple processing	Moisture sensitivity reduces ambient electrical stability	Charoonsuk et al. [50]
Chitosan	Quaternary ammonium modification	Smooth cast film	157	2.7	n.r	Enhanced surface potential and charge transfer behavior	Strong dependence on optimized hydrogen bonding to avoid brittleness	Zheng et al. [110]
Silk Fibroin	3D porous aerogel structuring	Ultralight porous aerogel	365	11.8	7.52 W/m^2^	Large specific surface area enhances contact electrification	Structural collapse may occur under prolonged cyclic compression	Tan et al. [7]
Silk Fibroin	MXene incorporation	SF@MXene composite film	418	11.6	9.92 W/m^2^	Synergistic interfacial charge trapping improves output performance	Complex processing required for uniform filler dispersion	Tan et al. [172]
Silk Fibroin	Natural crosslinking and MXene doping	Hierarchical porous film	748	n.r.	35.76 W/m^2^	Very high reported power density for single-electrode configurations	Multicomponent formulation complicates large-scale manufacturing	Wang et al. [173]

* n.r. indicates “not reported” in the referenced source/s.

**Table 12:** Advantages and limitations of selected biopolymers for TENG applications.

Biopolymer	Advantages	Limitations	Ref.
Cellulose	Abundant; tunable polarity; high mechanical stiffness	High porosity can reduce effective dielectric constant; neat cellulose has low intrinsic charge density	[1,4,34,54,69]
Chitosan	Biodegradable; antimicrobial properties; easily functionalized amine groups	High brittleness is a limitation on durability; high water affinity is a potential cause for deterioration in output in humid conditions	[22,89,99,106,115]
Silk Fibroin	Excellent biocompatibility; high tribopositivity; controllable degradation	β-sheet crystallization can cause nozzle clogging in AM; advanced formulations need complex composite processing	[2,3,7,172,190,197]

## Data Availability

No new data were created or analyzed in this study. Data sharing is not applicable to this article.

## Abbreviations

The following abbreviations are used in this manuscript:

0D	Zero-Dimensional (nanostructures with all dimensions at the nanoscale)
2D	Two-Dimensional
3D	Three-Dimensional
4D	Four-Dimensional
3DP	3D Printing
AC	Activated Carbon
AM	Additive Manufacturing
BaTiO_3_	Barium Titanate
BC	Bacterial Cellulose
BC/ZnO	Bacterial Cellulose/Zinc Oxide Composite
BAPO-OH	Bisacylphosphine Oxide-type Photoinitiator (water-compatible photoinitiator)
CB	Carbon Black
CEC	Cyanoethyl Cellulose
CMC	Carboxymethyl Cellulose
CMC-Na	Sodium Carboxymethyl Cellulose
CNC	Cellulose Nanocrystals
CNF	Cellulose Nanofibrils
CNT	Carbon Nanotube
CQ-TENG	Chitosan/Quartz Triboelectric Nanogenerator
CQAS	Chitosan Quaternary Ammonium Salt
CS	Chitosan
CS-AC	Chitosan/Activated Carbon Composite
CS/Plasticizer	Chitosan/Plasticizer Composite (e.g., with glycerol/PEG)
CS/Quartz	Chitosan/Quartz Fiber Composite
DC	Direct Current
DIW	Direct Ink Writing
DLP	Digital Light Processing
DMSO	Dimethyl Sulfoxide
EC	Ethyl Cellulose
ESE	Electrospray-Etching/Water Electrospray-Etching
FDA	Food and Drug Administration (U.S.)
FEP	Fluorinated Ethylene Propylene
GNPs	Graphene Nanoplatelets
hBN	Hexagonal Boron Nitride
HEC	Hydroxyethylcellulose
His	Histidine
HRP	Horseradish Peroxidase (enzyme used for enzymatic crosslinking)
LAP	Lithium Phenyl-2,4,6-Trimethylbenzoylphosphinate (photoinitiator)
m-CMC	Methacrylated Carboxymethyl Cellulose
MBA	N,N′-Methylenebisacrylamide (chemical crosslinker)
MCC	Microcrystalline Cellulose
MO-PPy	Modified Polypyrrole Composite
MXene	Two-Dimensional Transition-Metal Carbide/Nitride (MXene-type nanomaterial)
MWCNTs	Multiwalled Carbon Nanotubes
NaCl	Sodium Chloride
NaOH	Sodium Hydroxide
NC	Nanocellulose
PA6	Polyamide 6 (Nylon-6)
PANIs	Polyanilines
PCL	Poly(ε-Caprolactone)
PDMS	Polydimethylsiloxane
PDOTES	1H,1H,2H,2H-Perfluorodecyltriethoxysilane
PEDOT:PSS	Poly(3,4-ethylenedioxythiophene):poly(styrene sulfonate)
PEG	Polyethylene Glycol
PEO	Poly(ethylene oxide)
PLLA	Poly(L-Lactic Acid)
PPy	Polypyrrole
PTFE	Polytetrafluoroethylene
PVA	Poly(vinyl alcohol)
PVDF	Poly(vinylidene fluoride)
RMS	Root-Mean-Square
RSF	Regenerated Silk Fibroin
SF	Silk Fibroin
SF-MXene	Silk Fibroin/MXene Composite
SF-TENG	Silk Fibroin-Based Triboelectric Nanogenerator
SIL	Soft Imprinting Lithography
Sil-MA	Methacrylated Silk Fibroin (photocurable silk derivative)
SMP	Silk Microparticles
STENG	Silk-Based Triboelectric Nanogenerator (e.g., silk aerogel STENG)
SWCNTs	Single-Walled Carbon Nanotubes
TBAH	Tetrabutylammonium Hydroxide
TECD	Triboelectric Charge Density
TENG	Triboelectric Nanogenerator
UV	Ultraviolet
ZIF-8	Zeolitic Imidazolate Framework-8 (metal–organic framework)
**Nomenclature**	
*I_sc_*	Short-Circuit Current
*R_a_*	Root-Mean-Square Surface Roughness
*V_oc_*	Open-Circuit Voltage
wt%	Weight percentage/percentage by weight
